# Surface-elastic hydrogels delay senescence via the modulation of redox homeostasis and cytoskeletal tension

**DOI:** 10.1038/s41598-025-04779-7

**Published:** 2025-07-01

**Authors:** Thasaneeya Kuboki, Satoru Kidoaki

**Affiliations:** Laboratory of Biomedical and Biophysical Chemistry, Institute for Materials Chemistry and Engineering, Kyushu University, 744 Moto-Oka, Nishi Ku, Fukuoka, Japan

**Keywords:** MSC aging, Cellular senescence, Surface-elasticity-tunable gelatinous hydrogel, Redox homeostasis, Cytoskeletal tension, Delay senescence, Stem cells, Ageing

## Abstract

The Bone marrow-derived mesenchymal stem cells (MSCs) are widely used in clinical applications owing to their therapeutic properties. However, in vitro expansion of MSCs in tissue culture dishes induces aging, which reduces their quality through an undefined mechanism. This study delineates the role of substrate stiffness as a potential modulator to delay MSC aging by elucidating the senescence progression of preconditioned and serially passaged MSCs on engineered stiffness-tunable gelatinous hydrogels. We demonstrated that mechanoactivation of MSCs increased their radical-scavenging capacity, maintained redox homeostasis, restored actin dynamics, and maintained their therapeutic properties. The hydrogels alleviated hydrogen peroxide-induced oxidative stress, linking mechanical signaling to redox balance and senescence. These hydrogels restored actin remodeling, highlighting the importance of cytoskeletal tension and dynamics in cellular senescence. We established a new culture method to maintain the stemness, proliferation, motility, and osteogenic differentiation potential of MSCs by serially passaging the cells on stepwise surface-elastic gels. Evidence points toward the complex interplay between mechanical memories and actin dynamics and their implications for autophagic activity in the delaying of senescent MSCs via hydrogels. Our findings suggest that mechanoregulation of culture substrates finely tunes the balance between cellular stress, redox homeostasis, and cytoskeletal dynamics to delay the progression of MSC senescence.

## Introduction

Cellular senescence is defined as irreversible cell cycle arrest after prolonged cultivation^1^. This process is considered a hallmark of aging and is triggered by various signals and different kinds of stressors, such as oxidative stress, chemicals, irradiation, and replicative exhaustion^2,3^. Senescent cells exhibit typical phenotypes, such as changes in morphology to a flat, and irregular shape with higher levels of senescence-associated β-galactosidase (SA-β-GAL) activity and upregulation of cell cycle inhibitors such as p21 and p53^4^.

Redox homeostasis and cytoskeleton (CSK) integrity are key targets for interventions in the progression of aging and age-related diseases^5^. The balance between reactive oxygen species (ROS) and antioxidants is crucial for normal cell physiology and contributes to aging and pathology, partly by affecting cytoskeletal integrity^6^. CSKs, including actin fibers, microtubules (MTs), and intermediate filaments, play critical roles in proliferation, migration, and mitochondrial signaling^7^. In senescent cells, redox imbalance increases mitochondrial dysfunction and ROS accumulation^8^, and dysregulate actin and MT dynamics^9,10^.

Bone marrow-derived mesenchymal stem cells (MSCs) are widely used in clinical applications owing to their therapeutic properties. However, in vitro expansion of MSCs in tissue culture dishes (TC) induces replicative senescence (RP), which reduces their quantities and qualities^11^. MSCs acquire common characteristics through a plethora of triggers that decrease their proliferation and differentiation potencies^12^. Several studies have reported that MSC senescence can be reversed by modulating the redox balance using antioxidants or by reducing cytoskeletal tension using cytoskeletal tension inhibitors; however, these chemicals also have detrimental effects on cells during long-term usage^13,14^.

In vitro expansion is unavoidable in preparing a sufficient number of MSCs for therapeutic applications. To overcome these problems, technologies using biomaterials with stiffness that mimic the native microenvironment to maintain stem cell quality have been developed. Soft materials can modulate stem cell redox balance^15^ and delay senescence^16–19^, suggesting that biomaterials may serve as promising and safe tools to maintain MSC homeostasis. However, the underlying mechanism by which substrate stiffness affects senescence progression, particularly in the context of redox regulation and cytoskeletal tension, has not yet been well characterized, and the design of biomaterials with a rejuvenation capacity to maintain the therapeutic potential of MSCs remains largely unexplored.

In our system, the stiffness-tunable gelatinous gels that mimic native tissues can be fabricated and used for several applications, such as directional cell migration^20–22^ and stem cell manipulation^23^. Substrate stiffness significantly impacts MSC CSK organization and expression, affecting cellular tension and redox modulation. Proteomic analysis showed upregulation of antioxidant enzymes and key signaling molecules on gels, along with downregulation of CSK-related proteins^23^. Upregulation of antioxidant genes and neurogenic markers in stem cells on soft gels confirmed the influence of substrate stiffness on redox regulation and stem cell properties^24^.

This study aimed to delineate the role of substrate stiffness as a potential modulator of delayed aging of MSCs. The effects of mechanical stimuli on redox balance, CSK properties, and senescence progression were investigated in MSCs cultured on soft (S, 3–5 kPa), moderately stiff (M, 8–10 kPa), and stiff or hard (H, 20–40 kPa) hydrogels. MSCs preconditioned on hydrogels exhibited lower production of mitochondrial ROS and decreased expression of senescence markers. Stress-induced premature senescence (SIPS) was performed using hydrogen peroxide (H_2_O_2_) to induce oxidative stress in MSCs cultured on gels and TC. The hydrogels attenuated the effects of H_2_O_2_-induced oxidative stress and suppressed MSC senescence whereas silencing a redox regulator, nuclear factor erythroid 2-related factor (NRF2) amended these effects. MSCs on the hydrogels maintained the redox balance by increasing the expression of cytoprotective and longevity-related genes to scavenge ROS.

The hydrogels restored the actin remodeling of MSCs, as investigated using an actin-stabilizing agent while treating the cells on TC with an antioxidant and Rho kinase (ROCK) inhibitor reduced senescence. These results highlight the interplay among cytoskeletal tension-mediated mechanical signaling, redox homeostasis, and cellular senescence.

MSCs possess mechanical memories of the underlying substrates that they have experienced in the past, which bias their fate determination^25^. In our study, in addition to the application of single-stiffness hydrogels for MSC expansion, we manipulated the mechanical memories of the cells by allowing them to experience a gradual increase in the stiffness of the substrates. In comparison to single-stiffness conditions, serially passaged MSCs on gels with stepwise surface elasticity from soft to moderate stiffness and hard (SMH) gels showed the best performance in terms of maintaining their quantity and quality. These culture conditions enhanced cell motility, improved the proliferation and osteogenic differentiation potentials of aged MSCs and could maintain the function of older cells better than TC. To understand the underlying mechanism better, we investigated the response of Yes-associated protein (YAP)^26^, to varying substrate elasticities. YAP activation involves nuclear translocation, where it regulates genes for proliferation, differentiation, and cellular behavior. Nuclear YAP levels increase with extracellular matrix stiffness^26^ and decrease in aging cells^27^, serving as a marker of mechanical memory^25^. After serially passaging the MSCs on the hydrogels and switching to TC, we evaluated the ratio of nuclear to cytoplasmic YAP localization (N/C) and observed their stiffness-dependent mechanical memories, which correlated with the osteogenic differentiation potential. We examined other regulators that may be responsible for delaying senescence and observed the impact of substrate stiffness on alterations in actin dynamic regulators and autophagy-related genes.

Taken together, our findings demonstrate the role of compliant substrates in delaying MSC senescence phenotypes.

## Results

### Surface-elasticity-tunable gelatinous hydrogels could delay the senescence progression of MSC

**Fig. 1:**
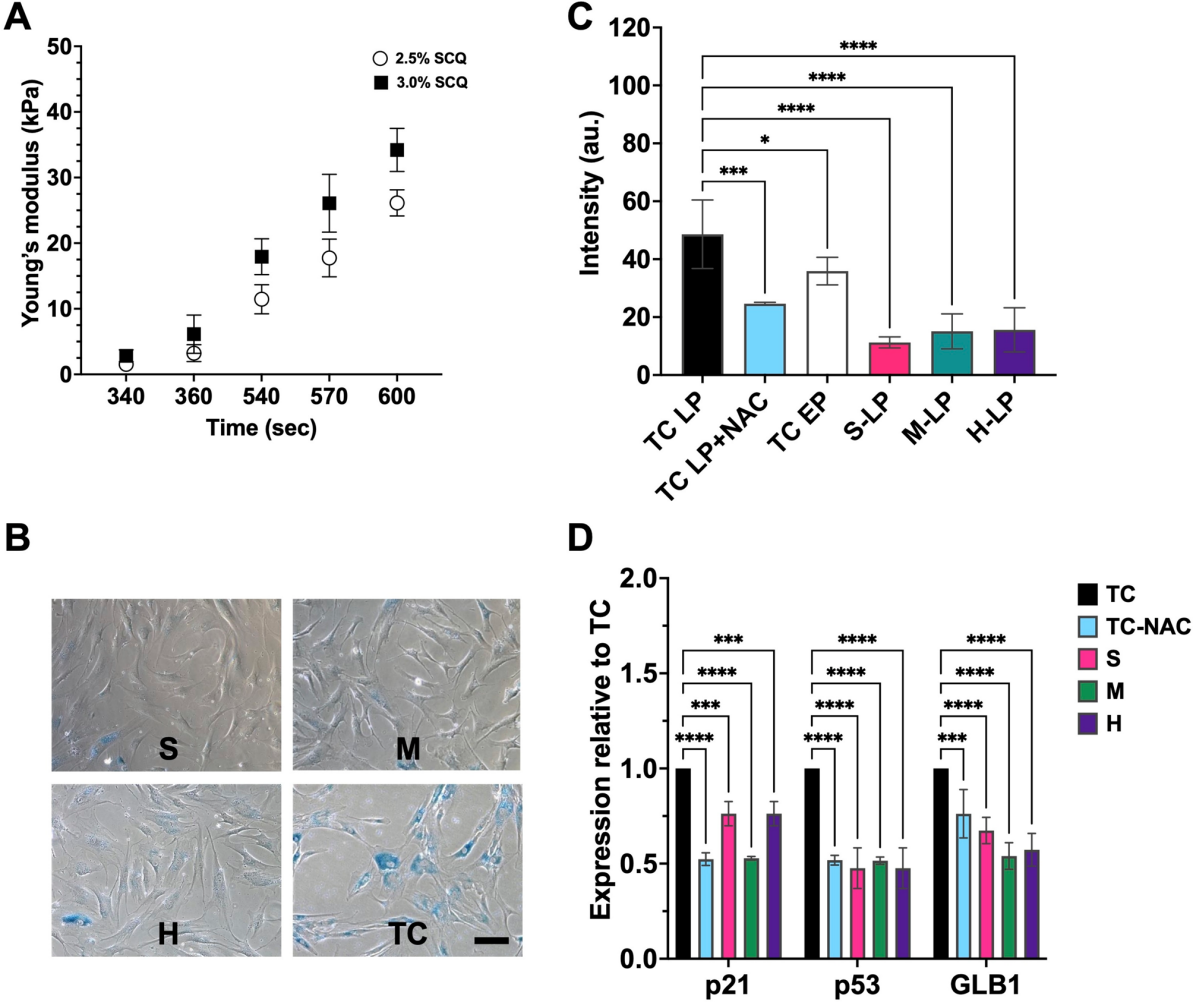
Preconditioning of the MSCs on surface-elasticity-tunable gelatinous hydrogels could delay senescence. (**A**) The young modulus of surface-elasticity-tunable gelatinous hydrogels prepared using LED illumination with two different concentrations of the photoinitiator (2.5 and 3% SCQ). (**B**) The SA-β-GAL staining images of the LP MSCs on S, M, H, and TC. The scale bar is 200 μm. (**C**) The SPiDER-β-GAL fluorescent intensity of EP, LP, antioxidant (NAC)-treated LP on TC, and the LP MSCs on gels. (**D**) The senescence gene expression of the MSC preconditioning on various substrate elasticities NAC-treated cells relative to TC. The statistical differences are shown (*p < 0.05, **** p < 0.0001, NS = no significant difference).

The surface elasticity of the gelatinous gels fabricated using LED illumination increased with exposure time and photoinitiator concentration, as evaluated by atomic force microscopy. (Fig. 1A). To study how aged MSCs interact with mechanical substrates, late-passage (LP) MSCs were seeded on TC, S, M, and H gels, and senescence was assessed by staining with SA-β-GAL (Fig. 1B). Compared with TC, MSCs preconditioned on gels, irrespective of surface elasticity, showed noticeably weaker staining for SA-β-GAL. Quantitative analysis was performed using a SPiDER-GAL fluorogenic probe and flow cytometry (Figs. 1C and S1). Early-passage MSCs (EP) exhibited low fluorescence intensity. The positive control antioxidant, N-acetyl cysteine (NAC), reduced the fluorescence signal in LP MSCs on TC compared with untreated cells. Similar to the NAC-treated cells and in agreement with the SA-β-GAL staining results, all gel conditions significantly decreased the SPiDER-GAL fluorescent signal of the MSCs compared with that of the TC.

Substrate elasticity reduced expression of senescence markers (galactosidase β-1, p21, and p53) in MSCs on gels and in NAC-treated cells compared to TC. (Fig. 1D). No significant gene expression differences were found among cells on S, M, and H gels. A TC coated with styrenated gelatin (StG) was included to assess the effect of a much stiffer substrate on the MSC senescent state. The cells on the TC coated with 0.3% StG decreased senescence but the suppression was less than that of the hydrogels (Figs. S2A–C and S3A).

These data demonstrate that, compared with TC, hydrogels can delay the senescence progression of MSCs.

### Surface-elasticity-tunable hydrogels could maintain redox homeostasis and attenuate the stress-induced oxidative stress of MSCs

**Fig. 2:**
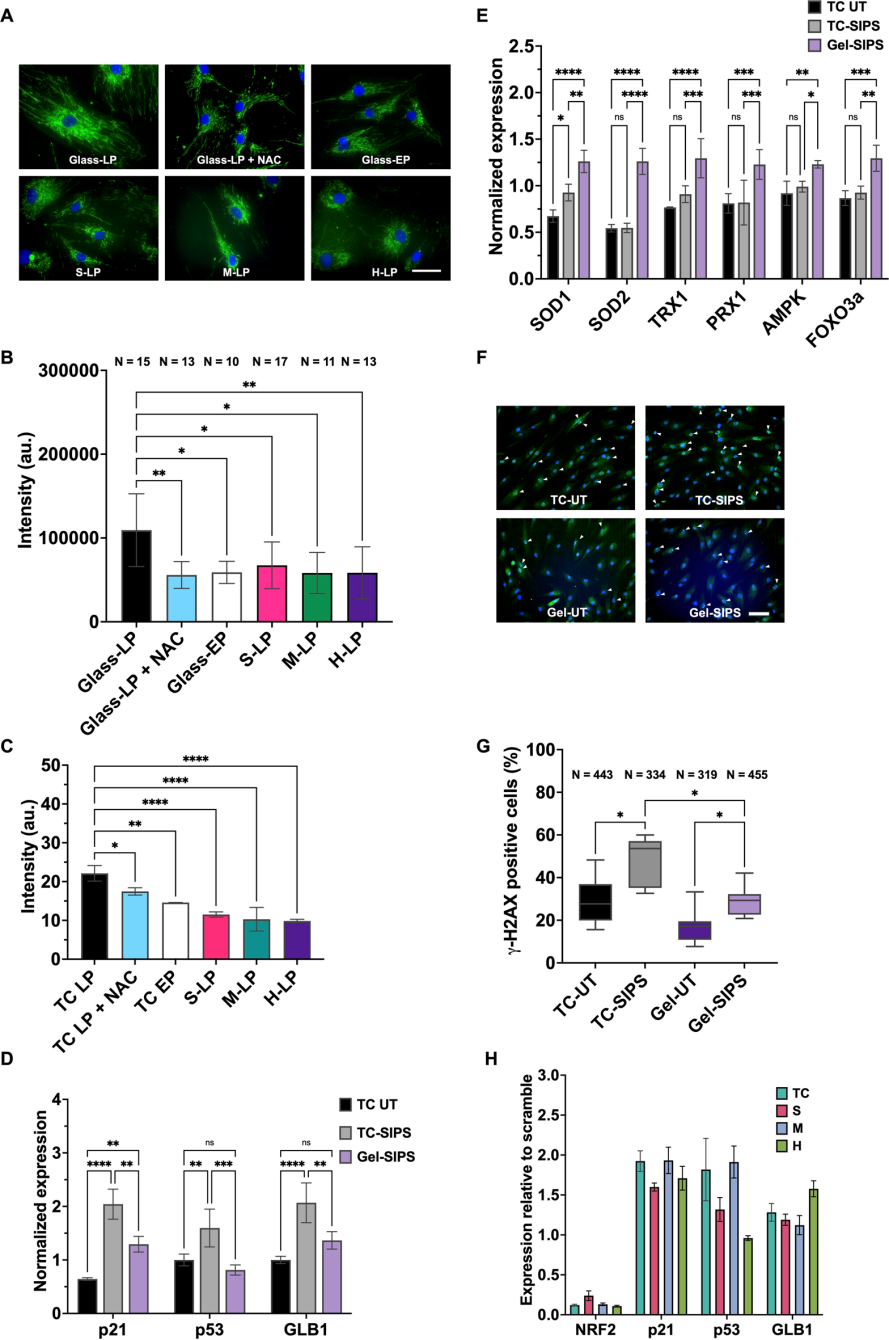
Surface-elasticity-tunable gelatinous hydrogels could modulate the redox balance and attenuate oxidative stress in the MSCs. (**A**) The MitoBright Green fluorescent staining images of the EP, LP, NAC-treated LP on TC, and the LP MSCs on various substrate elasticities. The scale bar is 50 μm. (**B**) Quantification of the MitoBright Green fluorescent signal at perinuclear regions. (**C**) The MitoSox Red staining images of EP, LP, NAC-treated LP on TC, and the LP MSCs on various substrate elasticities. (**D**) The senescence gene expression of the H_2_O_2_-induced oxidative stress (SIPS) in MSCs on gels, TC, and untreated control on TC (TC-UT). (**E**) The antioxidant, superoxide dismutase 1 and 2 (SOD1, 2), thioredoxin 1 (TRX1), peroxiredoxin 1(PRX1) and longevity-related, AMP-activated protein kinase (AMPK) and forkhead box O 3a (FOXO3a) gene expression of the SIPS in MSCs on gels, TC, and untreated control on TC. (**F**) The immunofluorescence staining images of γ-H2AX (green) and DAPI (blue) of the SIPS MSCs on gels and TC. The representative DNA damage foci are indicated (white arrows), with the quantification of positive cells (**G**). The scale bar is 100 μm. (**H**) The gene expression of the NRF2 knockdown MSCs on gels and TC. The statistical differences are shown (*p < 0.05, ** *p* < 0.005, *** *p* < 0.0005, **** p < 0.0001, *NS* no significant difference).

We examined how mechanical stimuli impact redox regulation in aged MSCs by analyzing mitochondrial changes and ROS production. On the rigid substrate, the LP MSCs showed intense staining for mitochondrial accumulation surrounding the nuclear and cytoplasmic areas (Fig. 2A). No remarkable difference in the staining pattern was observed between the young MSCs and hydrogels. The LP MSCs on the rigid substrate contained significantly higher fluorescence intensity than those on hydrogels and the NAC-treated group (Fig. 2B). LP MSCs showed a higher mitochondrial ROS fluorescence than EP MSCs, and the ROS levels decreased after NAC treatment (Figs. 2C and S4). Hydrogels significantly decreased mitochondrial ROS production in LP MSCs. On the StG-coated TC, the ROS level also decreased but less than on hydrogels (Fig. S3B,C).

To gain insight into the effect of mechanical stimuli on redox homeostasis, oxidative stress was induced using H_2_O_2_, and the cellular stress response and senescence were evaluated. In the SIPS control experiment, the treatment of MSCs with H_2_O_2_ highly upregulated NRF2, antioxidant genes, and senescence markers (Fig. S5). Because all hydrogels, irrespective of surface elasticity, decreased ROS production and suppressed senescence, a H gel was selected as a model substrate for further evaluation. Compared with TC, the hydrogels attenuated the effects of H_2_O_2_-induced oxidative stress by partially suppressing senescence (Fig. 2D). SA-β-GAL showed stronger staining on TC than on gels after oxidative stress induction (Fig. S6). The stress response, cytoprotective and longevity- related genes were strongly upregulated in SIPS MSCs on gels (Fig. 2E). Cells on hydrogels showed fewer DNA damage foci, based on γ-H2AX staining (Fig. 2F,G). Co-localization of γ-H2AX with p53 binding protein 1was more prominent in cells on TC than on gels. (Fig. S7). To confirm the association between redox homeostasis and senescence, gene silencing of NRF2 was performed. The results suggested that NRF2 knockdown enhanced MSCs senescence of on both gels and TC (Figs. 2H and S8), accompanied by compensatory upregulation of other alternative stress response molecules (Fig. S9).

### CSK tension and senescence

**Fig. 3:**
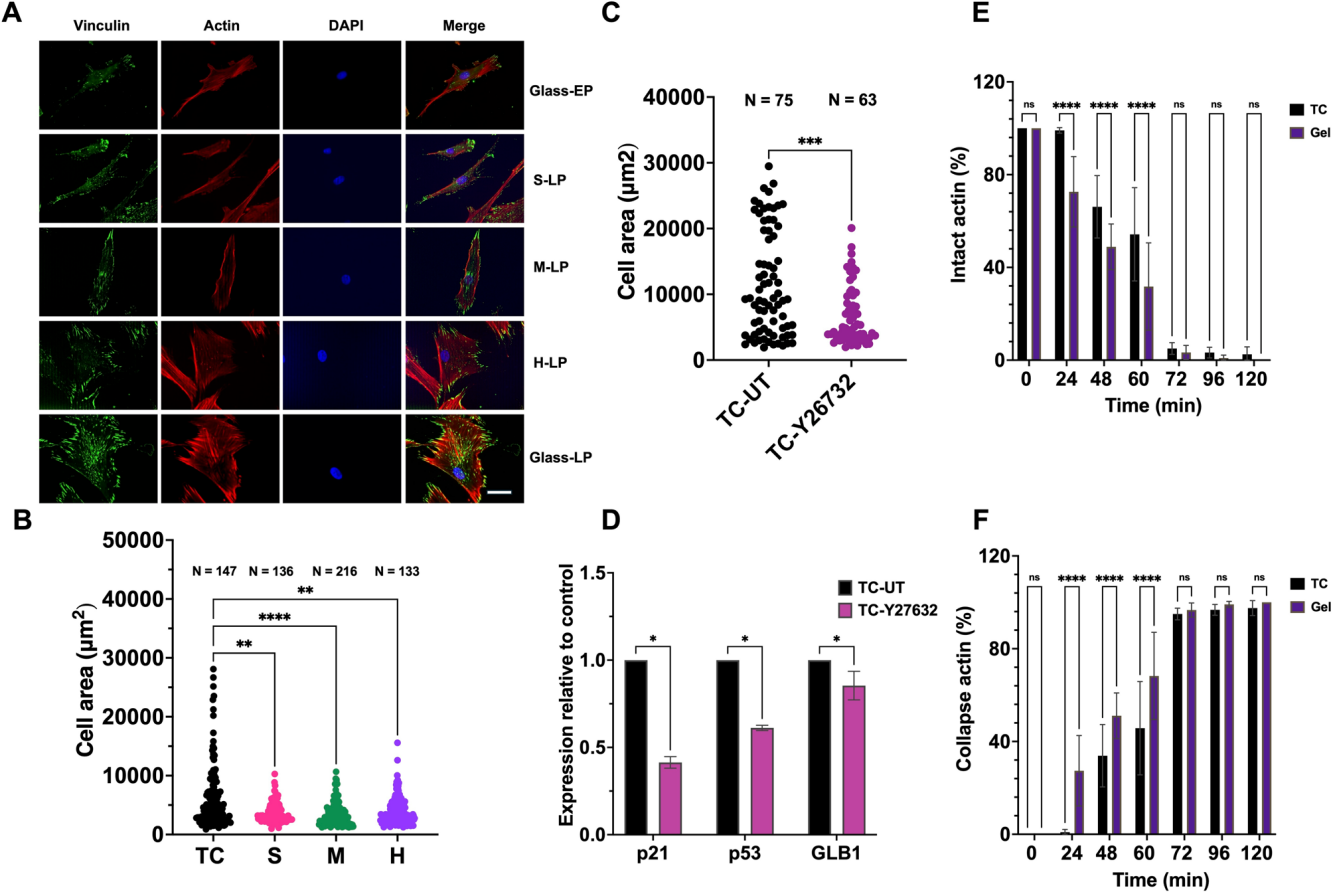
Surface-elasticity-tunable gelatinous hydrogels could modulate the cytoskeletal tension and dynamics of the MSCs. (**A**) The immunofluorescence staining images of vinculin, actin, and DAPI staining of the EP, LP MSCs on rigid control substrate compared with that on the gels. The scale bar is 50 μm. (**B**) The quantification of the cell area of the MSCs on various substrate elasticities. (**C**) The quantification of the cell area of TC-untreated (TC-UT) and Y-26732-treated MSCs. (**D**) The senescence gene expression of the control and Y-26732-treated MSCs. The percentage of the cells containing intact (**E**) and collapsed (**F**) actin structures after JAS treatment is shown. The statistical differences are shown (*p < 0.05, ** *p* < 0.005, *** *p* < 0.0005, **** p < 0.0001, *NS* no significant difference).

The involvement of cytoskeletal tension in MSC senescence was further investigated. LP MSCs showed altered cytoskeletal structure compared to EP MSCs (Fig. 3A), with increased spreading, larger size, abundant vinculin, and thick stress fibers. In contrast, LP MSCs on hydrogels had less vinculin and thinner actin fibers. The hydrogels significantly decreased the cell area compared with TC (Figs. 3B and S10). Treatment of MSCs with the ROCK inhibitor Y-26732 decreased the cell size (Figs. 3C and S11) reduced SA- and SPiDER-GAL (Fig. S12A–C), and downregulated senescence markers (Fig. 3D).

To confirm the significant impact of CSK dynamics on senescence, aged MSCs were cultured on TC and model substrate H gels for four days and transferred to TC for an additional two days before treatment with jasplakinolide (JAS) at various time points. In EP MSCs, actin collapse was noticeable after a short incubation period with JAS (Fig. S13). These results suggest that the actin dynamics of MSCs on TC were retarded, as demonstrated by the delayed response to JAS (Figs. 3E,F and S14). After 60 min of drug exposure, more cells on TC retained intact actin structures compared to those the gels. In contrast, actin remodeling of cells on the hydrogels was restored, as illustrated by the enhancement of actin collapse at 24 min. These findings suggested that the hydrogels reduced cell size, actin stress fibers formation and focal adhesions, indicating decreased cytoskeletal tension of MSCs. Inhibitor treatment significantly affected cytoskeletal tension and dynamics during cellular senescence.

### Serial passaging of the MSCs on the hydrogels delayed senescence

**Fig. 4:**
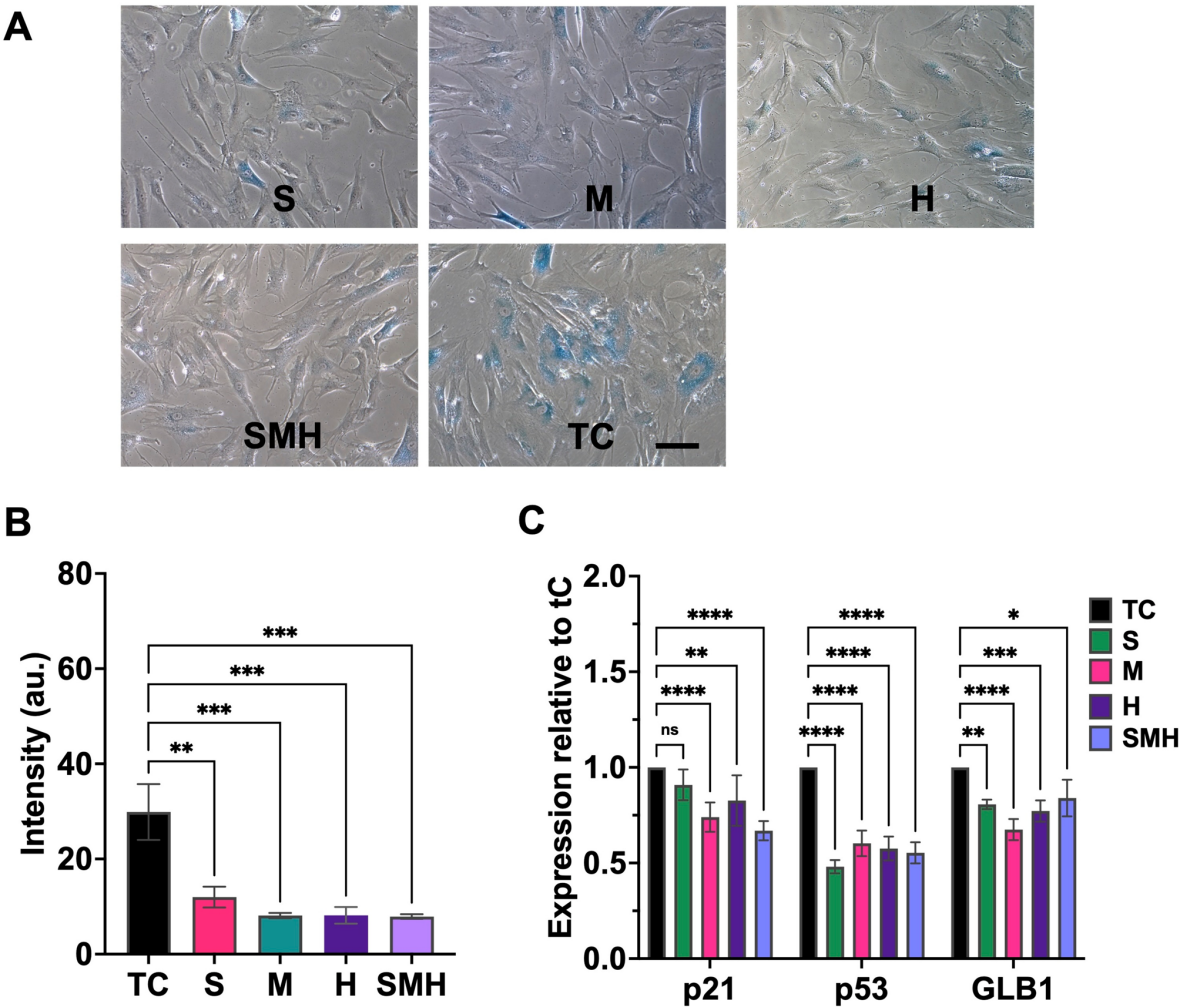
Long-term passaging of the MSCs on surface-elasticity-tunable gelatinous hydrogels could delay senescence. (**A**) The SA-β-GAL staining images of the LP MSCs on S, M, H, SMH. and TC. The scale bar is 100 μm. (**B**) The SPiDER-β-GAL fluorescent intensity of LP MSCs serially passaged on gels and TC. (**C**) The senescence gene expression of the MSC expansion on various surface-elastic substrates relative to TC. The statistical differences are shown (*p < 0.05, ** *p* < 0.005, *** *p* < 0.0005, **** p < 0.0001, *NS* no significant difference).

Thus far, the results suggest that preconditioning of MSCs on compliant hydrogels can maintain redox homeostasis, reduce cytoskeletal tension, increase actin remodeling, and suppress senescence. To confirm the properties of the hydrogels for the long-term maintenance of MSCs, the cells were serially passaged on TC, S, M, H and SMH gels. In agreement with the results obtained from the preconditioning experiments, the hydrogels showed reduced SA-(Fig. 4A) and the SPiDER-β-GAL staining (Figs. 4B and S15) and downregulated the senescence markers compared with the TC (Fig. 4C).

### Serial passaging of the MSCs on the hydrogels maintained the therapeutic potencies

**Fig. 5:**
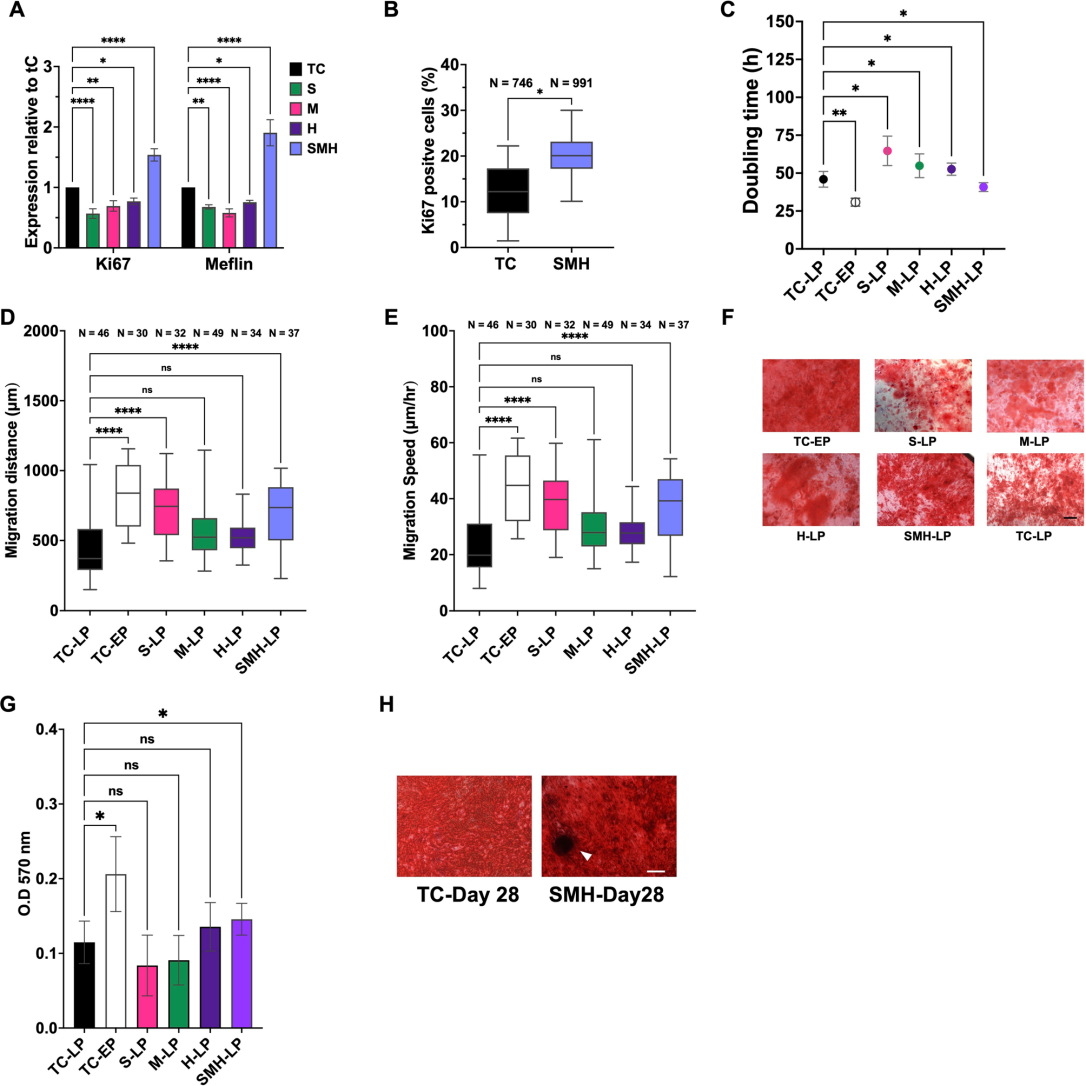
Long-term passaging of the MSCs on surface-elasticity-tunable gelatinous hydrogels could rescue the MSC therapeutic properties. (**A**) The Ki67 and Meflin expression of the MSC expansion on various substrates relative to TC. (**B**) The quantification of Ki67-positive cells from immunofluorescence staining of the MSC passaged on SMH gels and TC. (**C**) The doubling time of the LP MSCs serially passaged on TC, various substrate stiffness gels, and EP MSCs on TC. (**D**) The migration distance and velocity (**E**) of the MSC expansion on SMH and TC and reseeded on TC for time-lapse observation. (**F**) The Alizarin Red S staining images of EP MSCs on TC, LP MSC expansion on SMH and TC, and reseeded on TC at 14 days post-induction and the OD at 570 nm of the solubilized calcium matrix (**G**). – (**H**) The bone nodule formation at 28 days post-induction is indicated with a white arrow. The scale bar is 200 μm. The statistical differences are shown (*p < 0.05, ** *p* < 0.005, *** *p* < 0.0005, **** p < 0.0001, *NS* no significant difference).

We further investigated the ability of the hydrogels to maintain the therapeutic potencies of aged MSCs. In the RP, MSCs showed decreased expression of the proliferation marker Ki67 and the stemness marker meflin^28,29^ (Fig. S16), whereas serial passaging of MSCs on SMH hydrogels upregulated both genes (Fig. 5A). Immunofluorescence staining of Ki67 protein confirmed that SMH gels significantly promoted cell proliferation (Fig. 5B). In TC, the doubling time (DT) increased from ~ 30 h for EP to ~ 46 h for LP MSCs (Fig. 5C). Culturing LP cells on the single stiffness gels extended DT to 50–60 h. LP cells from SMH had a shorter DT (~ 40 h) than TC cells, but still longer than young cells. Serial passaging of MSCs on each substrate stiffness condition did not alter the expression of the MSC surface marker Stro-1 (Fig. S17).

The motility of the MSCs decreased with an increasing number of passages (Fig. S18). The results suggest that the migration distance (Fig. 5D) and speed (Fig. 5E) of the MSCs from the S and SMH hydrogels were significantly enhanced compared with those recovered from TC. The difference in motility between LP cells under both gel conditions and EP cells on TC was negligible.

We examined the effect of hydrogels on the osteogenic differentiation potential of MSCs, as the cells lost this property upon aging (Fig. S19). The results suggested that the MSCs from the S gels exhibited a tendency toward weak osteogenic differentiation potential in comparison to the stiffer substrates (Fig. 5F,G). The calcium production of MSCs from the H and SMH gels was higher than that of the TC-LP gel, whereas only the latter showed a significant difference. The results showed clear formation of bone nodules 4 weeks post-induction of MSCs from the SMH gels (Fig. 5H). Adipogenic induction was also performed but no apparent differences were observed (Fig. S20).

**Fig. 6:**
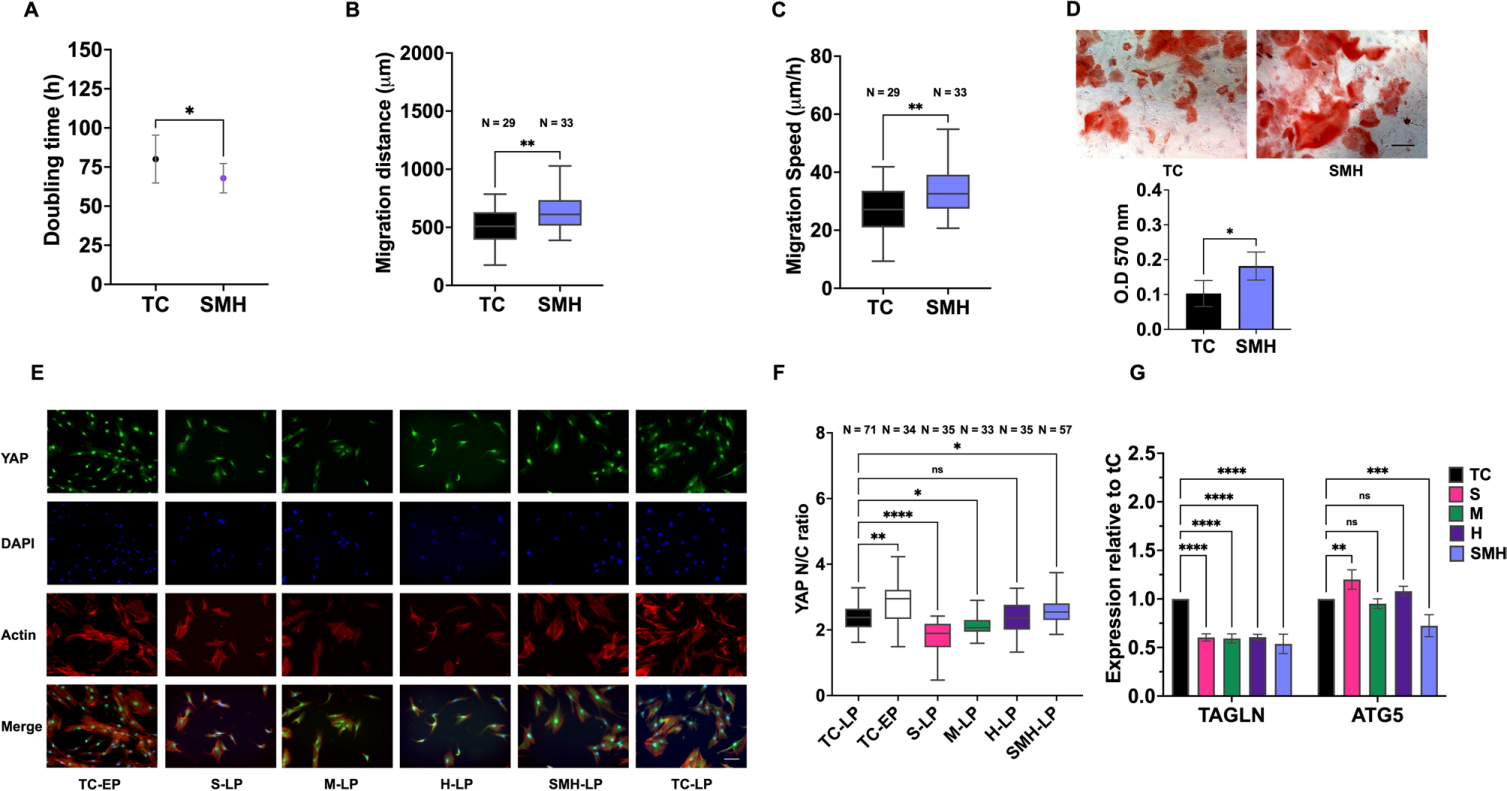
Long-term passaging of the MSCs on surface-elasticity-tunable gelatinous hydrogels could delay the senescence of the much older cells, modulate their mechanical memories, and influence actin polymerization regulators and autophagy-related genes. (**A**) The doubling time of the old MSCs serially passaged on TC and SMH gels. (**B**) The migration distance and velocity (**C**) of the MSC expansion on SMH and TC and reseeded on TC for time-lapse observation. (**D**) The immunofluorescence staining images of YAP, actin, and DAPI staining of the EP MSCs on rigid control substrate in comparison to LP MSCs collected from gels and TC. The scale bar is 100 μm. The quantification of the corresponding N/C ratio is shown in **F**. (**G**) The TAGLN and ATG5 expression of the MSC expansion on various substrates relative to TC. The statistical differences are shown (*p < 0.05, ** *p* < 0.005, *** *p* < 0.0005, **** p < 0.0001, *NS* no significant difference).

To address the question of whether the compliant hydrogels could rescue the therapeutic properties of the much older cells, we further investigated the influence of the SMH gels on later-passage MSCs (P20), as this gel condition showed the best performance in maintaining MSC properties. The DT of aged cells on TC remarkably increased to ~ 80 h, whereas that on SMH decreased to approximately 67 h (Fig. 6A). SMH gels increased the motility of MSCs compared to TC (Fig. 6B,C). Consistent with the results observed in younger passages (P16), MSCs from SMH hydrogels had a higher level of calcium production (Fig. 6D).

Immunofluorescence staining of YAP was performed (Fig. 6E) and the N/C ratio is shown in Fig. 6F. As seen in other findings, LP MSCs on TC had a significantly lower N/C ratio than EP cells. Our results suggest that MSCs from single-stiffness gels retain mechanical memory from their corresponding substrate stiffness. MSCs from S gels, with the lowest N/C ratio, showed even nuclear and cytoplasmic fluorescence. This ratio increased in the MSCs from M gels and reached a comparable level between the H gels and TC. Only cells from SMH gels showed a significantly higher N/C ratio than TC, with no significant difference compared with the EP MSCs.

Next, we investigated the mechanoregulation of transgelin (TAGLN), an actin-binding protein that modulate the cells mechanical properties, and actin dynamics^30^. Both YAP and TAGLN regulate the actin cytoskeleton, thereby influencing cell shape and motility^31,32^. In the RP, TAGLN expression increased with an increasing number of MSC passages (Fig. S21) and was downregulated in MSCs from all hydrogels compared with TC (Fig. 6G).

Autophagy maintains homeostasis, and its imbalance drives cellular senescence. We further explored the potential regulators responsible for the difference in MSCs from SMH gels by focusing on autophagy genes. We observed upregulation of the autophagy protein 5 (ATG5) in RP (Fig. S21). Compared with TC, ATG5 in the S gel sample slightly increased, while M and H gels showed no difference. Only MSCs from a SMH gels had significantly decreased ATG5 expression (Fig. 6G), indicating its potential role in cellular maintenance.

## Discussion

Cells retain mechanical memory, which can affect plasticity and fate^25^. Rigid TC surfaces may bias cell function and promote RP. Substrate stiffness was first recognized as a key property in several studies, including MSC fate determination^33^; however, its application in aging research remains limited.

Recent research has highlighted the potential of biomaterials to delay senescence^16–19^, but fundamental understanding of how compliant substrates achieve this remains limited due to MSC heterogeneity, the complexity of cellular senescence, and challenges in fabricating scalable biomaterials for long-term in vitro expansion. Polyethylene glycol diacrylate hydrogels functionalized with various adhesive ligands were used to identify substrate properties that support MSC proliferation, enhance paracrine factor secretion, and reduce RP, though further optimization is needed for large-scale therapeutic production^18^. A recent study showed that pH-responsive drug release hydrogels promoted intervertebral disc regeneration, reduced senescence, and alleviated pain in rat models^19^. While these studies highlight the potential of biomaterials in aging research, they have primarily addressed bulk matrix stiffness, where the elastic modulus of the hydrogel is modulated at the macroscopic scale and often measured using shear rheometry or theoretical estimates. These works did not examine surface elasticity, which describes the mechanical resistance of the hydrogel’s nanometric interfacial layer in direct contact with the cells. In contrast, our study explicitly investigates this surface-specific property and its biological impact to offer a novel mechanobiological perspectives.

Efficient control of MSC aging and rejuvenation is crucial for improving health and lifespan. Understanding these mechanisms will aid in developing better anti-aging strategies and provide insights into age-related diseases. The previous foundational studies have provided key mechanistic insights into how hydrogel properties affect MSC behavior and aging phenotypes. Inspired by these important contributions, our work investigates surface elasticity as an additional, previously underexplored dimension of the cell–material interaction, offering a more localized mechanotransductive perspective. Our study demonstrated the capacity of surface-elasticity-tunable gelatinous hydrogels to delay the senescence phenotypes of MSCs. We employed AFM–based nanoindentation to quantify the elastic modulus at the hydrogel surface with nanometer-scale resolution. The LED illumination-induced gelation method generated cell-adhesive gels with a wide range of Young’s moduli that mimicked the native tissue. This simple fabrication technique allows scalable biomaterial production without extra steps for surface functionalization, making it ideal for large-scale MSC expansion in therapeutic applications. In a recent study, Young’s modulus of 0.1–0.5% gelatin-coated TC slightly decreased, but to a negligible value (~ 3 GPa), compared with that of non-coated TC (~ 5 GPa)^34^, which is still considered a rigid substrate. The 0.3% StG- coated TC in our study showed less senescence suppression than the hydrogels, suggesting that the soft mechanical microenvironment is a key factor in delaying senescence.

Matrix stiffness regulates mitochondrial morphology, dynamics, and localization^35^. Senescent cells have increased mitochondrial mass and accumulation of dysfunctional mitochondria^36^. Our results suggest that the hydrogels reduce mitochondrial accumulation, decrease mitochondrial ROS levels, and suppress senescence. Mitochondria move along actin via motor proteins, with MT guiding their transfer—processes that influence oxidative stress and aging. Compliant hydrogels may enhance mitochondria–cytoskeleton interactions by preserving cytoskeletal structure and redox balance.

To highlight the importance of the redox balance in the mechanoregulation of senescence, SIPS was performed to induce senescence. MSCs on hydrogels show less senescence progression than those on TC, suggesting better ROS-scavenging capacity for detoxifying exogenous H_2_O_2_. Low basal expression of NRF2 protein and compensatory responses limited our ability to clearly define its specific role. The similar senescence state in NRF2 knockdown cells under TC and gel conditions indicate that NRF2 is not a unique factor but one of several contributors to delayed senescence.

While NRF2 appears to play a role, the compensatory upregulation of stress-response genes suggests a more complex regulatory network. When NRF2 is knocked down, cells lose a key defense against oxidative stress and partially restore redox balance through alternative pathways (e.g. catalase, peroxiredoxin or thioredoxin), leading to upregulation of other stress-response molecules. Different substrate stiffnesses triggered distinct compensatory responses, highlighting the importance of redox balance in maintaining MSC homeostasis and delaying senescence. In particular, transcription factors such as forkhead box O transcription factor (FOXO)^37^, metabolic sensors like AMP-activated protein kinase (AMPK)^38^and thioredoxin (TRX)^39^, which were upregulated in our SIPS model on hydrogels, are strong candidates for further investigation in future mechanistic studies. AMPK activation enhances oxidative damage repair, while FOXO3a regulates stress responses, longevity, and activating antioxidant genes like superoxide dismutase and TRX to counteract oxidative damage. The upregulation of these genes in SIPS MSCs on gels suggests their involvement in antioxidant regulation, helping the cells adapt to oxidative stress. Further research on their interconnections will enhance our understanding of the redox and mechanoregulation of senescence.

Aging cells become stiffer and lose the ability to rearrange their CSKs in response to stimuli. Cytoskeletal tension is crucial for actin stress fiber integrity^40^. Upregulation of senescence-responsive stress fiber proteins was observed in RP cells with thick stress fibers^41^. Aged cells on the TC exhibited large focal adhesions and thick actin fibers, reflecting high cytoskeletal tension. Y-27632, a cytoskeletal tension inhibitor used for age-related diseases, reduced cell size and senescence marker expression, highlighting the role of abnormal tension in aging. RhoA, an upstream regulator of ROCK, activates ROCK upon binding and regulates cell morphology, migration, and autophagosome formation^42^. The RhoA/ROCK pathway controls actin remodeling and myosin contractility, increasing cytoskeleton stiffness^43^ Activation of this pathway promotes cellular senescence by modulating F-actin polymerization in response to mechanical cues^44^. This report agrees with our results, which show that the inhibition of RhoA/ROCK reduces CSK stiffness and cellular senescence, suggesting that this cellular state could be delayed or reversed by regulating cytoskeletal tension.

Aging of MSCs affects antioxidant activity and actin turn over^9^. In agreement with this study, aged cells with lower cytoskeletal dynamics responded to JAS in a delayed manner, as demonstrated by the higher ratio of cells with intact actin fibers and fewer collapsed actin fibers compared with young cells. The response of cells on the gels was similar to that of young cells, indicating that actin remodeling in MSCs on the gels was rescued. These results highlighted the importance of cytoskeletal tension and dynamics in cellular senescence.

The final and most important goal was to demonstrate the rejuvenation capacity of the hydrogels in rescuing the therapeutic properties of MSCs. MSCs serially passaged on hydrogels showed significantly suppressed senescence and reduced ROS without affecting Stro-1 expression, consistent with previous studies that the MSC surface marker remains unchanged with increasing passage^45^ and after long-term culture on gels^16^. Long-term cultivation of aged MSCs on a single-stiffness hydrogel could not promote proliferation, which is consistent with other findings that soft substrates reduce MSC proliferation^18,46^.

MSC motility is crucial for the efficacy of cell-based therapies^47^. Decreased ROS defense and reduced actin dynamics in aged MSCs can affect their function, including motility^9^, thereby reducing their regenerative potential. In our study, although the single-stiffness hydrogels could not support cell proliferation, soft hydrogels can restore cell motility to a level comparable to that of young cells. Further optimization of the balance between maintaining proliferation and promoting other beneficial properties of MSCs in a soft microenvironment will make a significant contribution to the field of regenerative medicine.

Biomaterials that maintain both the proliferation and differentiation potential of MSCs are critical for future therapeutic applications. In this study, we established culture conditions using SMH gels to rescue proliferation potential, maintain stemness, and enhance the motility of MSCs. Compared with other substrates, only SMH gels significantly increased meflin expression. Meflin is a marker of specific MSC subpopulations with enhanced regenerative potential, self-renewal, and differentiation^28,29^. Although the underlying mechanism remains unclear, the upregulation of meflin in MSCs derived from SMH gels offers several benefits for therapeutic applications.

We investigated the effect of the hydrogels on osteogenic differentiation, a critical therapeutic property of MSCs that is difficult to maintain with aging. Our findings suggest that the calcium production of MSCs from the S and M gels was slightly lower than that from the other samples. This is not unexpected, as these stiffness range are not suitable for support osteogenic differentiation^33^. The H gels with stiffness that favors osteogenesis showed increased calcium deposition compared with the softer substrates and slightly enhanced deposition compared with TC. SMH gels maintained the osteogenic differentiation potential, as demonstrated by the significantly increased calcium production and enhanced formation of bone nodules. We did not observe a remarkable difference in adipogenicity between cells from gels and TC, as in a previous report^16^. This discrepancy is possibly owing to donor-to-donor variations, or the different properties of the biomaterials and adhesive ligands being studied. We further confirmed the functional restoration capacity of SMH gels in older cells and observed a similar trend. Although DT cannot be reversed compared with young cells, SMH gels are still better than TC for maintaining the regenerative capabilities of MSCs, as their motility and osteogenic potential could be rescued.

The characteristics of the MSCs from SMH appeared to be different from the single stiffness H gels, indicating the mechanoactivation of distinct regulatory pathways for both culture conditions. Optimal cytoskeletal integrity is linked to lifespan and aging. In aged MSCs, altered chromatin structure silences genes for maintenance and repair^48^. Cytoskeletal tension influences chromatin structure, promoting relaxation and enhancing gene expression related to cell cycle progression. The mechanical memory is regulated by the epigenetic remodeling of chromatin, and mechanical amplitude can induce transcriptional memory, promoting MSCs proliferation^49^. In the SMH system, gradual modulation of contractile force and cytoskeletal tension and avoidance of the imprinting of mechanical memories from a single stiffness during expansion may contribute to improving MSC quality. The cytoskeletal tension-induced epigenetic modifications may explain the enhanced proliferation of MSCs on SMH gels, which requires further exploration.

We investigated the memory of the cells after serial expansion under each stiffness condition and transferred them to a rigid substrate. Nuclear YAP levels indicated that the cells remembered their previous physical environment. These memories were associated with their osteogenic differentiation potential, which was enhanced in MSCs from stiffer substrates with a higher N/C ratio and suppressed in samples with a low N/C ratio. The recovery of YAP activation in MSCs from the SMH gels supports the notion that exposing the cells to stepwise surface-elastic substrates avoids the imprinting of mechanical memory and improves their quality.

Reduced actin dynamics cause mitochondrial depolarization and increased ROS production^50^. TAGLN, a regulator of actin dynamics and stress response, is typically elevated in senescent cells^51^. We observed the downregulation of TAGLN in MSCs from hydrogels, emphasizing the pivotal role of CSK dynamics in delaying senescence. However, no significant differences were observed among the hydrogel samples, indicating that other factors are involved in the machanoactivation of the MSCs on the SMH gels.

The CSK mechanical properties influence the formation and movement of autophagosomes, which are associated with actin filament^52^. In senescence, autophagic activity is typically impaired; however, in some cases, elevated levels of autophagic activity have been observed in RP MSCs after long-term cultivation^53^. In our system, we observed the upregulation of ATG5 in RP cells, whereas this gene was downregulated only in SMH gels, indicating that the mechanoregulation of autophagy may contribute to increased MSC functional maintenance capacity. However, this must be proven through gain- and loss-of-function studies of specific autophagy genes on different substrate elasticities to delineate their involvement in delaying aging and promoting rejuvenation. The mechanism underlying the optimal functional maintenance capacity of SMH gels remains elusive and requires further investigation using global omics.

In summary, the results of this study suggest that compliant hydrogels can serve as suitable microenvironments for MSC culture. MSCs cultured on hydrogels possess a higher radical-scavenging capacity and can better maintain redox homeostasis, reduce cytoskeletal tension, and enhance the recovery of actin dynamics. Senescent phenotypes can be delayed or reversed by the long-term cultivation of MSCs on SMH hydrogels to maintain their therapeutic properties. Cytoskeletal tension and mechanical memory enhance cellular mechanosensitivity, helping MSCs respond to mechanical cues and maintain integrity to counteract senescence-related changes.

## Materials and methods

### Cell culture

Primary human MSCs (Lonza, Tokyo, Japan) at passages 3–33 were cultured in mesenchymal stem cell basal medium (Lonza). The cells were maintained on TC at 37 °C under 5% CO_2_ in a humidified incubator. The early passages (EP, P5–8), middle passages (P9–11), and late passages (LP, ≥ P12) were used for the evaluation of aging and rejuvenation in response to substrate stiffness. For the RP control, cells were cultured on TC for 3–4 days and serially passaged until they reached LP.

### Preparation of gelatinous gels with different surface elasticity

To prepare cell-adhesive hydrogels with different surface elasticities, photocurable StG is synthesized in-house, as previously described^20,21^. Briefly, a solution of phosphate-buffered saline containing StG (30% w/w; degree of derivatization: 100%) and water-soluble sulfonyl camphorquinone (2.5–3% w/v gelatin) was spread between a vinyl-silanized glass substrate and a poly(N-isopropylacrylamide) (Sigma Aldrich, St. Louis, MO) coated glass substrate. Gelation was induced by using an LED panel light (5.2 mW / cm^2^ at 488 nm; light source: LMG150 × 180NW; AITEC SYSTEM, Japan). The surface elasticity of each hydrogel was determined using microindentation analysis as previously described^20^. The force-indentation curves of the gel surface were measured using atomic force microscopy (JPK NanoWizard 4, JPK Instruments, Bruker Nano GmbH, Germany) with a commercial silicone-nitride cantilever with a nominal spring constant of 0.03–0.09 N/m (qp-BioAC-CI CB3, Nanosensors) at five randomly selected points (nine of 50 × 50 µm elasticity map) of at least three different samples (n = 3). The Young’s modulus of the surface was evaluated from the force-indentation curves of the gel surface by fitting it to the Hertz model^54,55^. For in vitro cultivation and expansion of MSCs on various surface-elastic substrates, the cells were cultured in Dulbecco’s modified Eagle’s medium (Gibco BRL, Grand Island, NY, USA) supplemented with 10% fetal bovine serum (Gibco BRL), 100 units/mL penicillin, and 100 μg/mL streptomycin. Control StG-coated TC was prepared by diluting StG in D.W. to obtain a final concentration of 0.3% (v/v) and sterilizing by filtration. The diluted solution was added to the 35 mm dishes and incubated at 37 °C for 30 min. The solution was discarded, and the dishes were air-dried for more than 2 h. StG-coated dishes were rinsed twice with phosphate-buffered saline prior to cell seeding.

A schematic of MSC cultivation under each substrate stiffness condition is shown in Fig. S22. For the preconditioning experiment, MSCs were seeded at a density of 4,000 cells/cm^2^ on TC, soft (S, 3–5 kPa), moderately stiff (M, 7–10 kPa), and stiff or hard gels (H, 20–40 kPa) for four days. The antioxidant-treated control group was prepared by replenishing the cells with fresh medium containing 1 mM NAC (FOCUS Biomolecules, PA, USA). For long-term in vitro expansion, MSCs were cultured on the substrates for four days and serially passaged on the corresponding substrates for an additional two cycles. Long-term expansion of the MSCs with increasing stiffness of the hydrogels, designated as the SMH condition, was performed by first culturing the cells on soft gels for four days and followed by serial passaging and culturing on moderate and hard gels in the last cycle. Cell proliferation was monitored using the CM30 incubation monitoring system (Evident). The images of the MSCs on each substrate condition acquired on days 2 and 4 post-seeding were used for cell counts using Fiji software, and the cell numbers obtained were used to calculate the DT^16^.

### Senescence-associated β-galactosidase staining

The degree of senescence was evaluated by staining with a chromogenic dye using a SA-β-GAL kit (Cell Signaling Technology, Danvers, MA, USA) and the fluorogenic probe SPiDER-β-GAL (Dojindo, Kumamoto, Japan), according to the manufacturer’s instructions. SPiDER-β-GAL provides greater sensitivity and increased specificity toward SA-β-GAL, as the staining procedure also includes bafilomycin A, which inhibits endogenous β-galactosidase activity. This method offers quantification capabilities along with reduced background noise and shorter incubation times. Color images of the SA-β-GAL-stained cells were obtained using a BZ-X700 (Keyence Corporation, Osaka, Japan) with a 20 × objective lens. The fluorescence signal of SPiDER-β-GAL-stained cells was evaluated using a flow cytometer (Guava EasyCyt Flow Cytometer; Millipore, Japan).

### Detection of mitochondrial ROS and mitochondria staining

MSCs were cultured on substrates with different stiffness for five days, and mitochondrial ROS staining was performed using MitoSoxRed (Invitrogen, Thermo Fisher Scientific). The cells were washed twice with phenol red-free-Hank’s Balance Salt Solution (Fujifilm Wako, Tokyo, Japan) and stained with 4 µM MitoSoxRed for 20 min. The stained cells were collected for flow cytometry.

For mitochondrial staining, the cells were washed twice with phenol red-free Leibovitz’s L-15 medium (Thermo Fisher Scientific) supplemented with 10% fetal bovine serum and antibiotics. The mitochondria of MSCs were stained with 25 nM MitoBrightGreen (Dojindo) diluted in L-15 medium. Nuclei were co-stained with NucBlue™ Live Ready Probes™ (Molecular Probes, Thermo Fisher Scientific) according to the manufacturer’s instructions. After 15 min of incubation, the samples were washed twice with the medium, and the fluorescent signal was observed in live cells with a structured illumination microscopy function using the 60 × oil objective lens of a BZ-X700 microscope. Mitochondrial intensity in the perinuclear region was quantified using Fiji software. NucBlue staining was used to identify the nucleus and to create a region of interest (ROI). The nuclei of MitoBright Green-stained cells were selected using NucBlue ROI. To quantify perinuclear fluorescence, ROIs were expanded by 15 pixels to measure the integrated density, which was subtracted from the intensity of MitoBright Green-stained nuclei.

### Induction of SIPS and evaluation of radical-scavenging capacity

SIPS was induced as previously described^56^. The cells were sparsely plated overnight and treated with 200 µM H_2_O_2_ for 2 h. The cells were washed twice with fresh media and cultured for four days. The cells were then reseeded and treated with H_2_O_2_ in a similar manner for two additional cycles. In this study, SIPS was used to evaluate the radical-scavenging capacity of MSCs in response to oxidative insults with different substrate stiffness values. MSCs were cultured on the substrates for four days before H_2_O_2_ treatment. The cells were washed and cultured in fresh media for four additional days before SA-β-GAL staining, sample collection for gene expression analysis, and immunofluorescence staining of DNA damage-related proteins.

### Inhibitor treatment

The MSCs were cultured on TC for five days in the presence of 2 µM ROCK inhibitor, Y-27632 (Sigma Aldrich). The cells were reseeded and cultured for two additional days without inhibitors for cell area measurement. For SA-β-GAL, SPiDER -β-GAL staining, and the gene expression analysis, the MSCs were cultured for two days and treated with 25 µM Y-27632 for four additional days before staining and RNA isolation.

Actin remodeling was evaluated using the actin-stabilizing agent jasplakinolide (JAS, Adipogen® LIFE SCIENCE, CA, USA) at a concentration of 250 nM. MSCs were maintained on TC and H gels for four days and reseeded on TC for two additional days. The cells were treated with JAS for 24, 48, 60, 72, 92, and 120 min before staining with rhodamine-phalloidin (Cytoskeleton Inc. Denver, Co., USA) and DAPI (Sigma Aldrich), following the protocols provided by the manufacturers. Fluorescent images of the cells were captured using a 20 × objective with a BZ-X700, and the number of cells containing collapsed and intact actin filaments was analyzed using the Fiji software (https://fiji.sc/).

### Measurement of cell area and evaluation of cell motility using time-lapse analysis

Changes in the morphology and motility of RP cells were investigated in the EP and LP MSCs. MSCs were cultured on the hydrogels and TC for four days for cell area measurement using the calcein-AM staining method. Cells were stained with calcein-AM dye (Invitrogen) according to the manufacturer’s instructions. Fluorescent images of the cells were captured using a 10 × objective with a BZ-X700, and the size of the area was quantified using Fiji.

Phase-contrast time-lapse analysis was performed on images obtained using a CM30 incubation monitoring system. For the gel experiment, MSCs were serially passaged on TC, S, M, H, and SMH gels and reseeded on TC for two days prior to time-lapse observation. The movies were recorded for 18 h at 15-min intervals. Cell trajectories were obtained by manually tracking the centers of the nuclei of moving cells using the MTrackJ plugin in Fiji to obtain the accumulated migration distance and velocity.

### Immunofluorescence staining

Immunofluorescence staining of MSCs cultured on substrates of various stiffnesses was performed as previously described^23^. The cells were incubated with primary antibodies: polyclonal anti-mouse; Ki-67 (Cell Signaling and Technology, Tokyo, Japan), polyclonal anti-rabbit; histone-H2AX pSer139 (γ-H2AX, Novus Biologicals, CO, USA), 53BP1 (Genetex, CA, USA), polyclonal anti-mouse; inculin (Santa Cruz Biotechnology Inc., CA, USA), polyclonal anti-rabbit; YAP1 (Genetex), secondary antibodies (donkey anti-rabbit conjugated with Alexa Fluor^@^488, donkey anti-mouse conjugated with Alexa Fluor^@^488, and donkey anti-rabbit conjugated with Alexa Fluor^@^568 (Invitrogen). Rhodamine-phalloidin was added to the statin F-actin filaments. The images were captured using a BZ-X700 microscope. Quantification of YAP nuclear and cytoplasmic ratios was performed using Fiji software. The N/C ratio was calculated using the following formula: (Nuclear YAP intensity/area)/(Cytoplasmic YAP intensity/cytoplasmic area). Immunofluorescence staining of Stro-1 was performed without membrane permeabilization step by incubating the cells with the primary antibody Stro-1 anti-mouse IgM (R&D System™, MN, USA) and the secondary antibody, goat anti-mouse IgM-FITC (Santa Cruz Biotechnology Inc., CA, USA).

### RNA expression analysis and gene silencing

RNA was isolated from MSCs that were preconditioned or serially passaged on various surface-elastic substrates using a Direct-Zol Microprep RNA purification kit (ZYMO RESEARCH, CA, USA). DNase digestion, reverse transcription, and quantitative real-time polymerase chain reaction (qPCR) were performed as previously described^24^. Reference genes hypoxanthine phosphoribosyltransferase and pumilio RNA-binding family member 1 were used to normalize gene expression. The gene silencing of NRF2 was performed using small interfering RNA. MSCs were cultured on the substrates for four days. small interfering RNAs, including scrambles (sc-37030, Santa Cruz Biotech) and NRF2 (sc-37030, Santa Cruz Biotech), were delivered into the cells using the Lipofectamine RNAiMax transfection reagent (Invitrogen) following the manufacturer’s protocol. After 24 h of incubation, the samples were collected for RNA isolation, qPCR analysis, and SA-β-GAL staining.

### Evaluation of differentiation potentials of the MSCs

The differentiation potential of LP MSCs was determined by serial passaging of the cells on TC, S, M, H, and SMH gels and reseeding on TC for two days before induction. Osteogenic induction was performed after the cells reached 70–80% confluency using an osteogenic supplement (PromoCell, Heidelberg, Germany) for two and four weeks. Calcium mineralization was stained using Alizarin Red S^57^, and phase-contrast color images were obtained using a BZ-X700 microscope. The calcium matrix was extracted using 10% cetylpyridinium chloride at 37 °C for 15 min. The supernatant was transferred to 96-well plates, and the absorbance was measured at 570 nm.

Adipogenic differentiation was induced by culturing the cells in Minimal Essential Medium (Fujifilm Wako, Tokyo, Japan) until 100% confluency. The induction was performed for two weeks using an adipogenic supplement from the Human Mesenchymal Stem Cell Functional Identification Kit (R&D System™). The lipid droplets were stained with Oil Red O, and phase-contrast color images were obtained using BZ-X100.

### Statistical analysis

All data were obtained from at least three independent experiments and are expressed as the mean standard deviation. A non-parametric Kruskal–Wallis test, followed by a post-hoc test, was conducted to evaluate cell area, motility, and actin remodeling. Ordinary two-way ANOVA followed by Dunnett’s multiple comparison test was performed for all qPCR comparison experiments. For comparison of the cell area of Y-26732-treated MSCs and immunofluorescence staining for γ-H2AX and Ki67, a non-parametric Mann–Whitney test was used. Statistical analyses of DT and Alizarin Red S quantification were performed using the Brown-Forsythe and Welch ANOVA tests.

## Supplementary Information

Supplementary Information 1.

Supplementary Video 1.

Supplementary Video 2.

Supplementary Video 3.

Supplementary Video 4.

Supplementary Video 5.

## Data Availability

The primer sequences used for qPCR in the current study are available from the corresponding author upon request. Supplementary figures and movie files are provided in the Supplementary Information.
