# Supplementary material for: Surface-elastic hydrogels delay senescence via the modulation of redox homeostasis and cytoskeletal tension
Source: Sci Rep. 2025 Jul 1;15:20460. doi: 10.1038/s41598-025-04779-7 (PMC12217731; doi:10.1038/s41598-025-04779-7)
Supplement: Supplementary file 1 — Supplementary Information 1. [file 41598_2025_4779_MOESM1_ESM.pdf]

Supplementary information

Surface elasticity hydrogels delay senescence via the modulation of redox homeostasis and cytoskeletal tension

Thasaneeya Kuboki* and Satoru Kidoaki

Laboratory of Biomedical and Biophysical Chemistry, Institute for Materials Chemistry and Engineering, Kyushu University, 744 Moto-oka, Nishi ku, Fukuoka, Japan

* Corresponding Author: kubokit@ms.ifoc.kyushu-u.ac.jp; tel.: 81-92-802-2505; fax: 81-92-802-2509

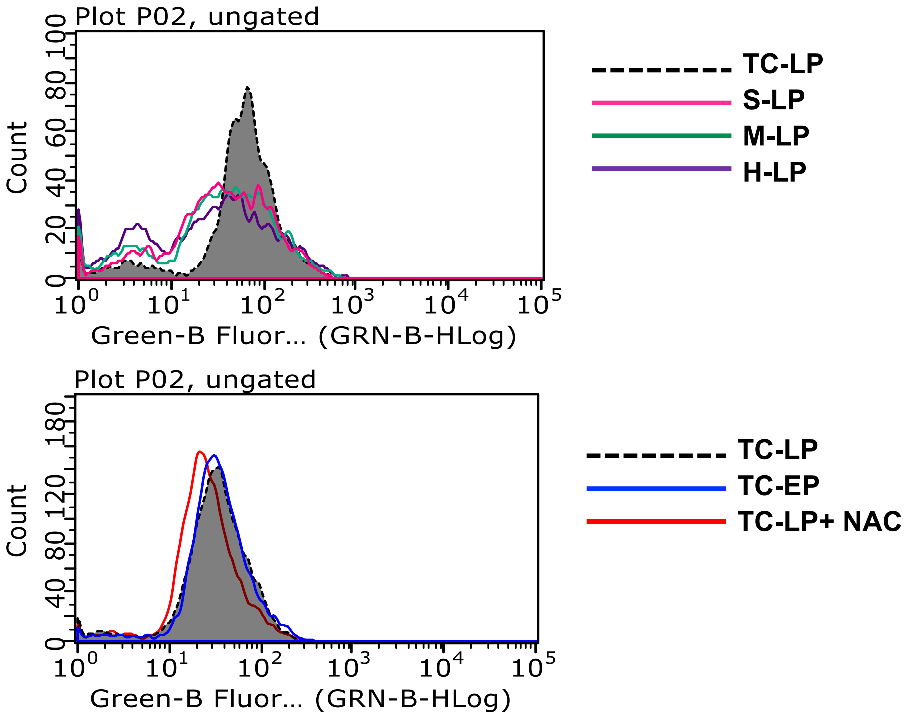

Figure S1 Flow cytometry analysis of SPiDER-β-GAL of EP, LP, antioxidant (NAC)-treated LP on TC, and the LP MSCs on gels.

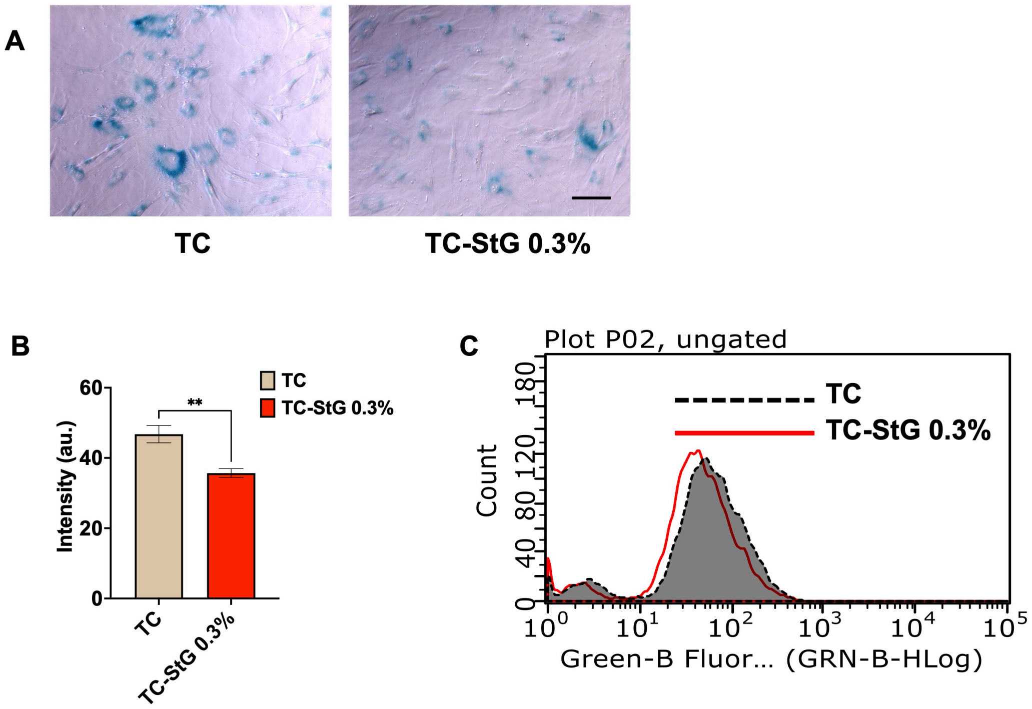

Figure S2 A. SA- β-GAL staining of MSCs preconditioned on 0.3% StG coated-TC (TC-StG 0.3%) and control non-coating (TC). The scale bar is 100 μm. **B** and **C** Flow cytometry analysis of SPiDER-β-GAL staining.

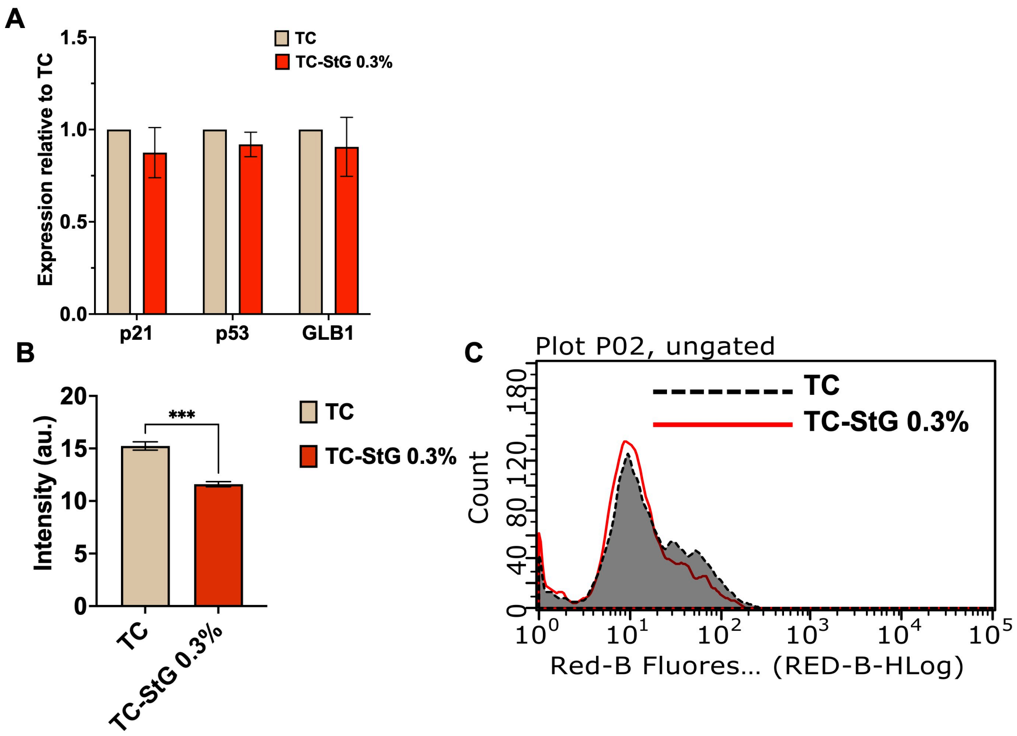

Figure S3 A. Senescence gene expression of MSCs preconditioned on TC-StG 0.3% and control non-coating. **B** and **C** Flow cytometry analysis of MitoSOX Red staining.

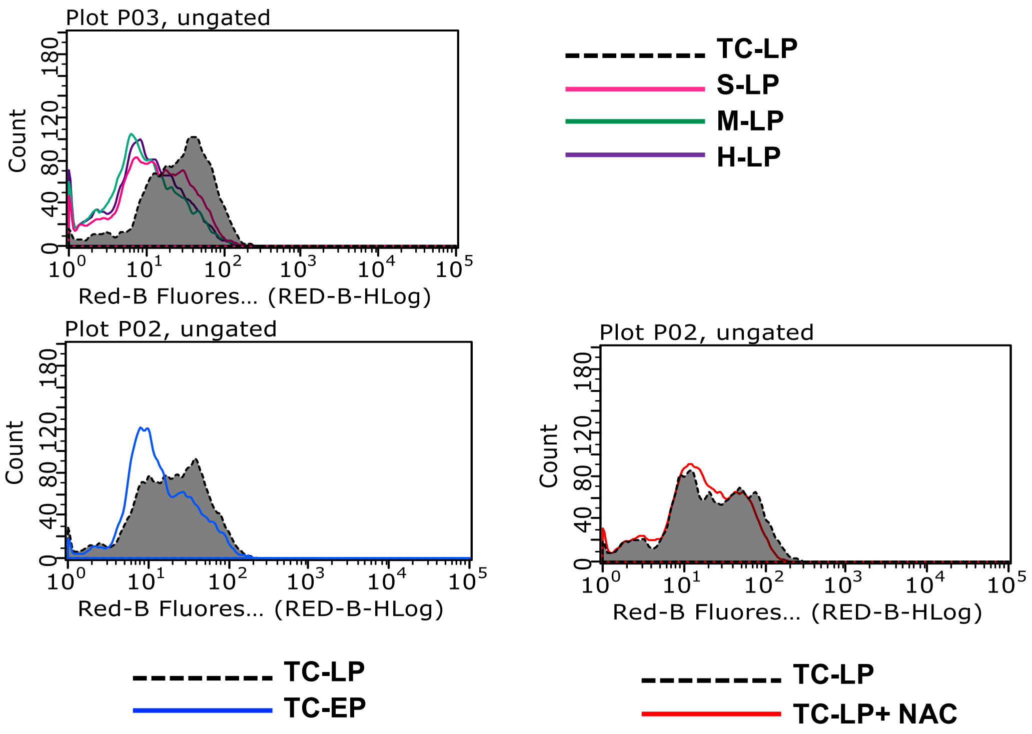

Figure S4 Flow cytometry analysis of MitoSox Red of the EP, LP, NAC-treated LP on TC, and the LP MSCs on gels.

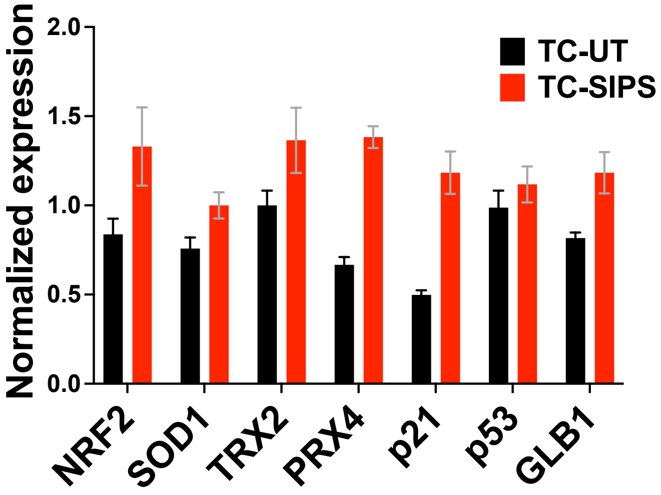

Figure S5 The redox regulator (NRF2), antioxidant (Superoxide Dismutase1 (SOD1), Thioredoxin2 (TRX2), Peroxiredoxin4 (PRX4)) and senescence gene expression of the H_2O_2 induced premature senescence (TC-SIPS) and the control untreated MSCs (TC-UT).

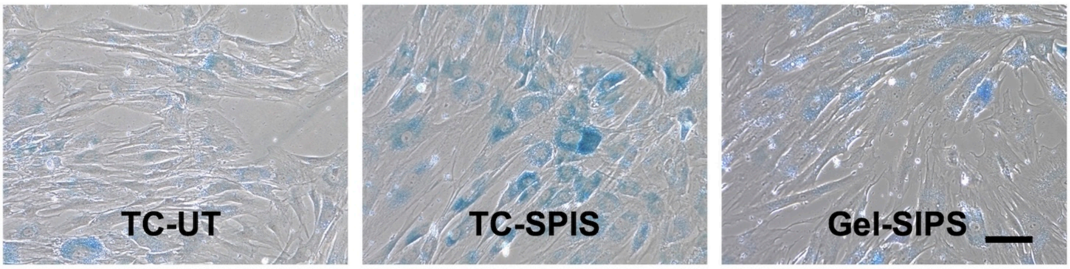

Figure S6 The SA-β-GAL staining images of the SIPS MSCs on TC (TC-SIPS) and gel (Gel-SIPS) compare with the untreated cells (TC-UT). The scale bar is 100 μm.

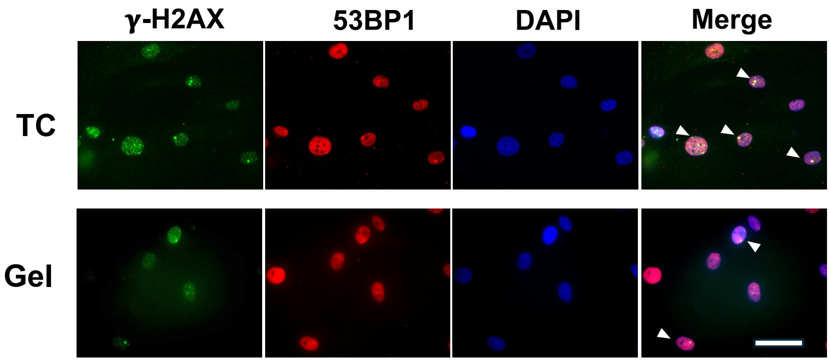

Figure S7 Immunofluorescence staining of γ-H2AX (green), 53BP1 (red) and DAPI (blue) of the MSCs on TC and gel. The example of DNA damage foci are indicated with white arrow heads. The scale bar is 50 μm.

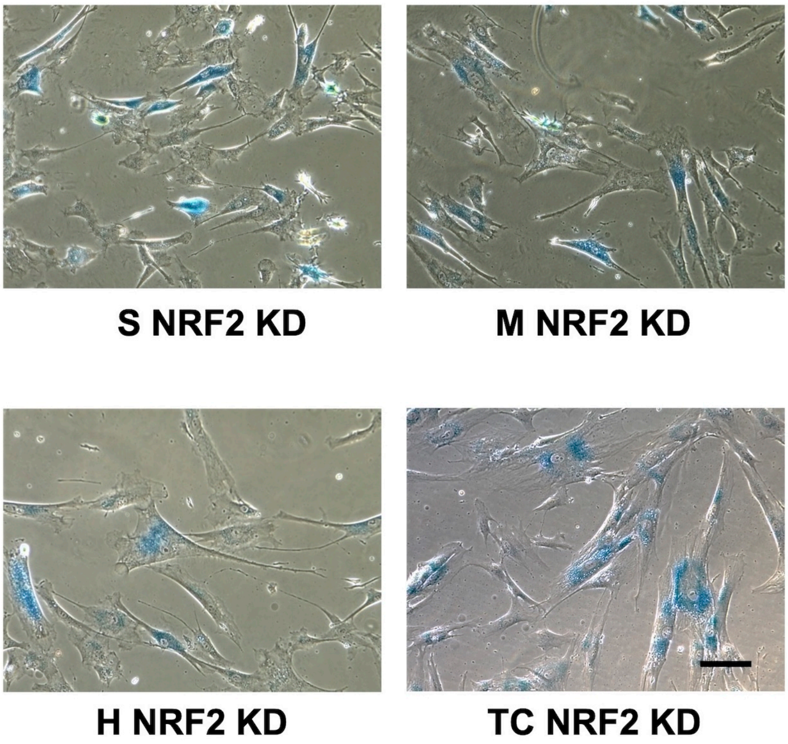

Figure S8 The SA-β-GAL staining images of the NRF2 KD-MSCs on TC and gel. The scale bar is 100 μm.

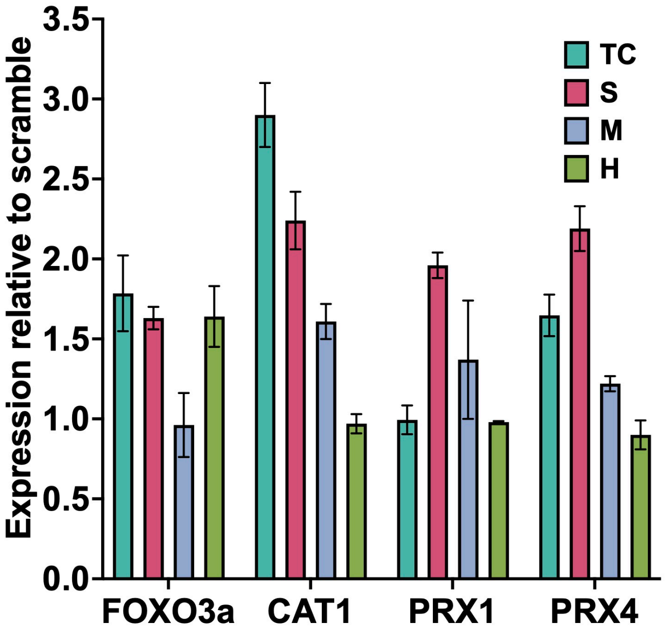

Figure S9 The alternative stress response molecule forkhead box O transcription factor 3a (FOXO3a), catalase 1 (CAT1), Peroxiredoxin 1 and 4 (PRX1, PRX4)) gene expression of the NRF2 knocked down MSCs on gels and TC.

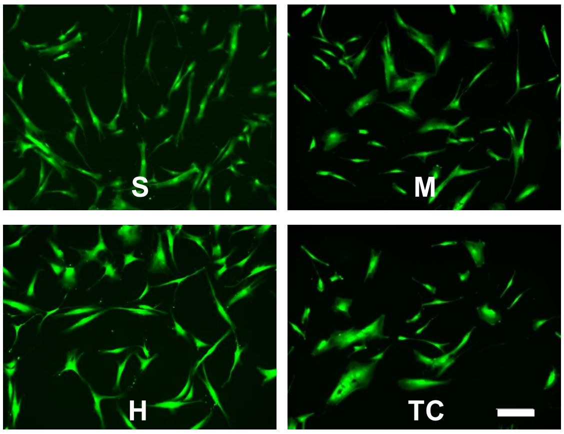

Figure S10 The calcein AM fluorescent staining of the MSCs on TC and gels. The scale bar is 200 μm.

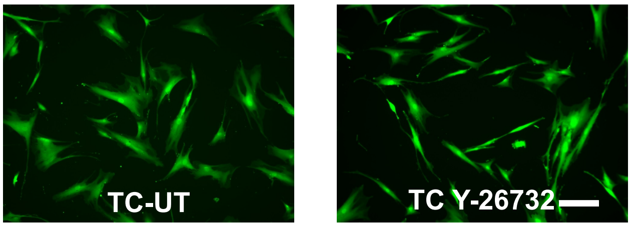

Figure S11 The calcein AM fluorescent staining of the MSCs on TC untreated (TC-UT) and Y-26732 (TC Y-26732) treated cells. The scale bar is 200 μm.

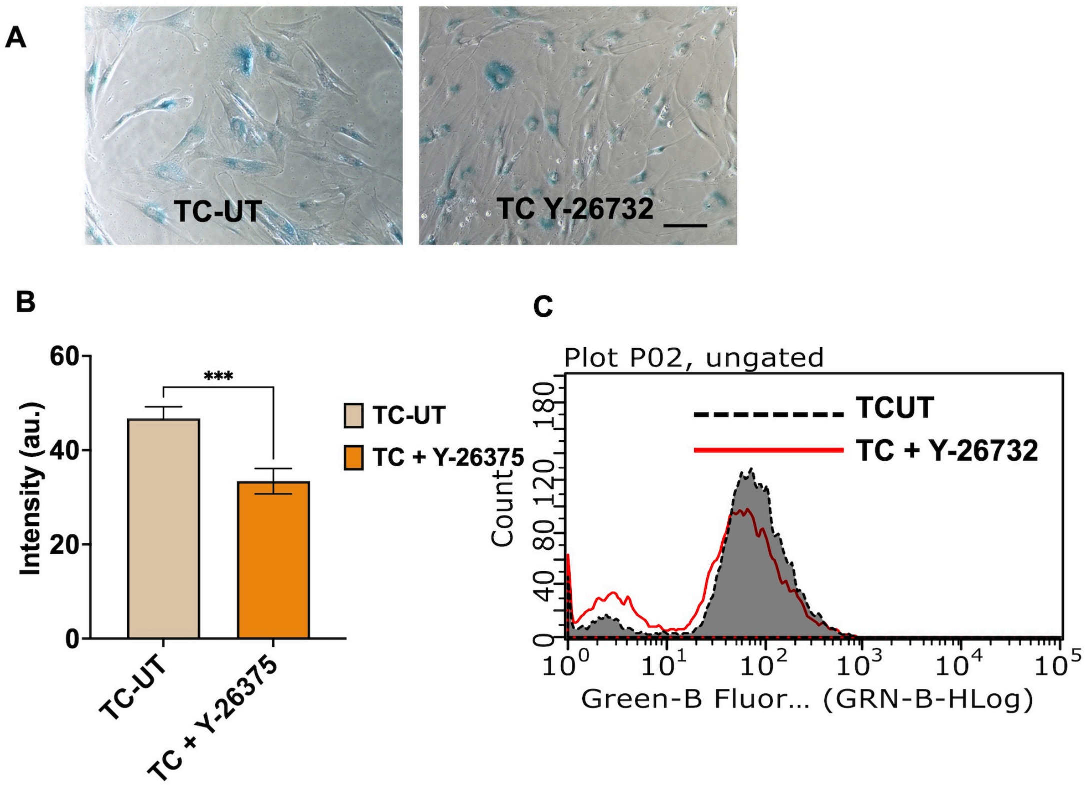

Figure S12 A. SA- β-GAL staining of Y-26732 treated MSCs and control untreated. **B** and **C** Flow cytometry analysis of SPiDER-β-GAL staining. The scale bar is 100 μm.

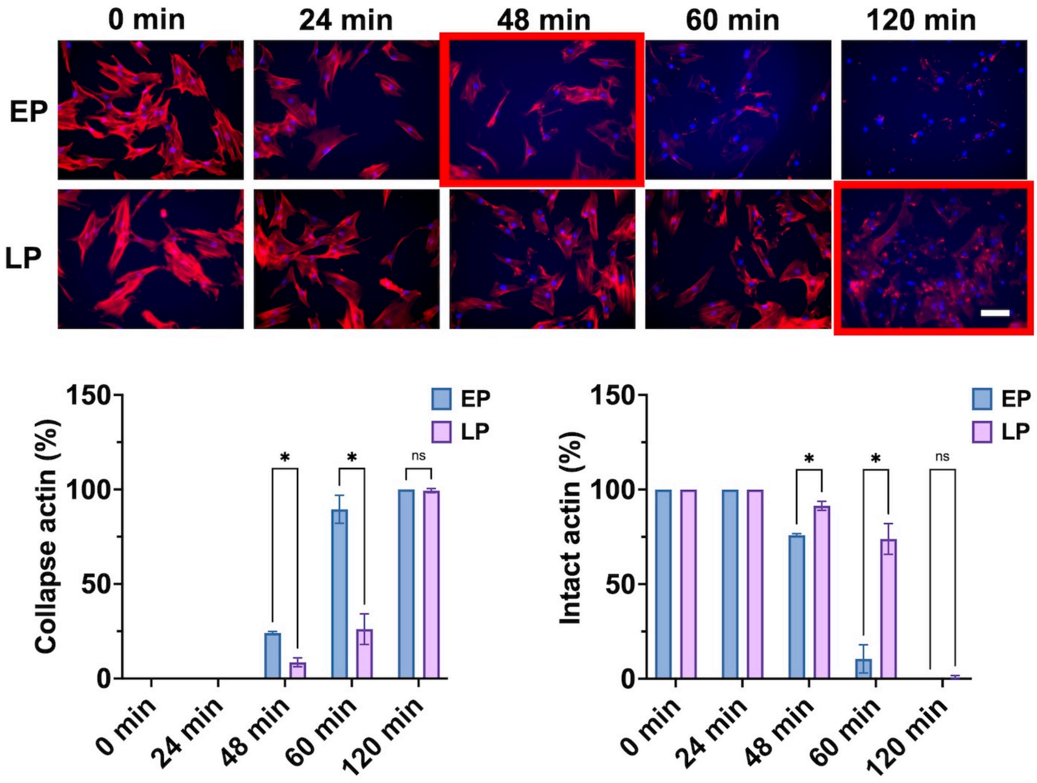

Figure S13 The fluorescent images of Rhodamine Phalloidin (red) and DAPI (blue) staining after JAS treatment of the EP and LP MSCs on TC (upper panel). The timepoints that showed remarkably high numbers of cells with collapse actin structure are highlighted with red boxes. The scale bar is 100 µm. The percentage of the cells containing intact and collapse actin after JAS treatment are shown (lower panel). The statistical differences are shown (*$p < 0.05$, NS = no significant difference).

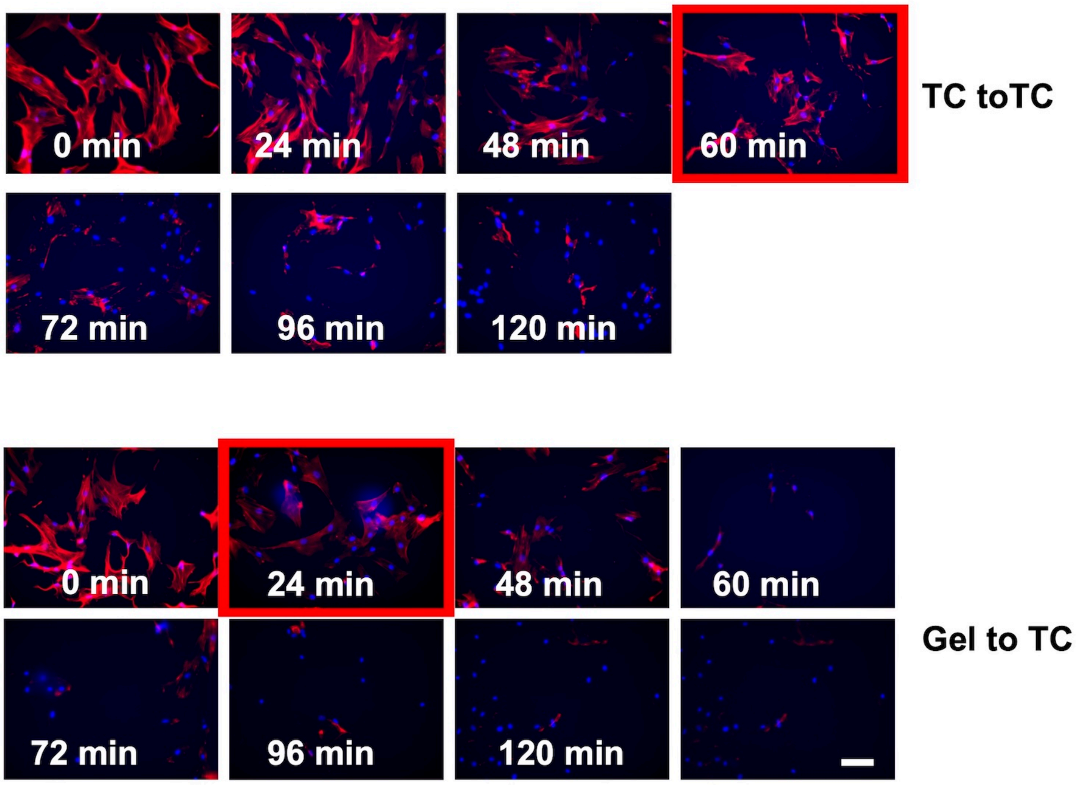

Figure S14 The fluorescent images of Rhodamine Phalloidin (red) and DAPI (blue) staining after JAS treatment of MSCs maintained on TC and gels and reseeded on TC. The timepoints that showed remarkably high numbers of cells with collapse actin structure are highlighted with red boxes. The scale bar is 100 μm.

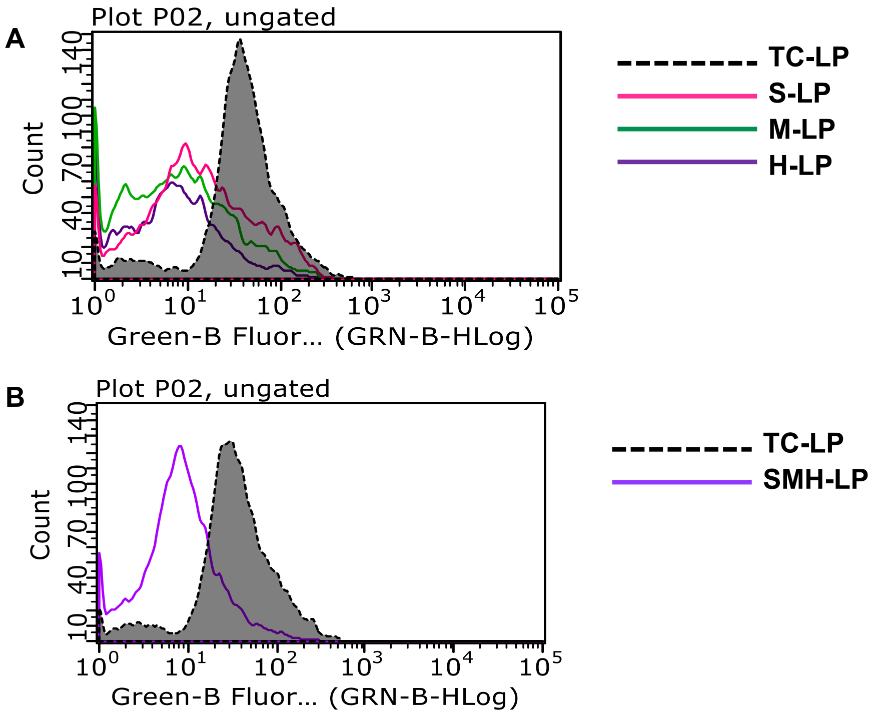

Figure S15 Flow cytometry analysis of SPiDER-β-GAL of LP MSCs serially passaged on gels and TC.

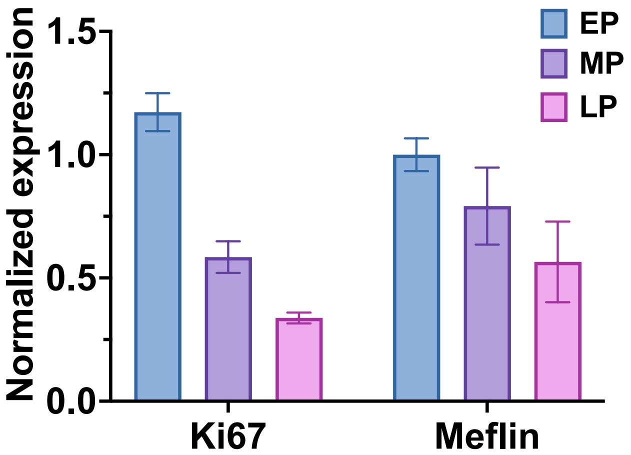

Figure S16 The expression of proliferation marker Ki67 and stemness marker Meflin in young and RP MSCs.

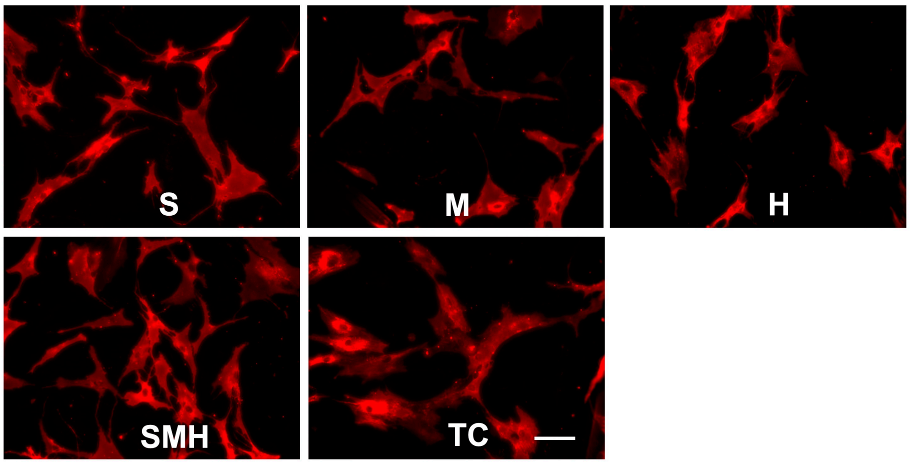

Figure S17 Immunofluorescence staining of the MSCs collected from TC, S, M, H, SMH gels and re-seeded on TC. The scale bar is 100 μm.

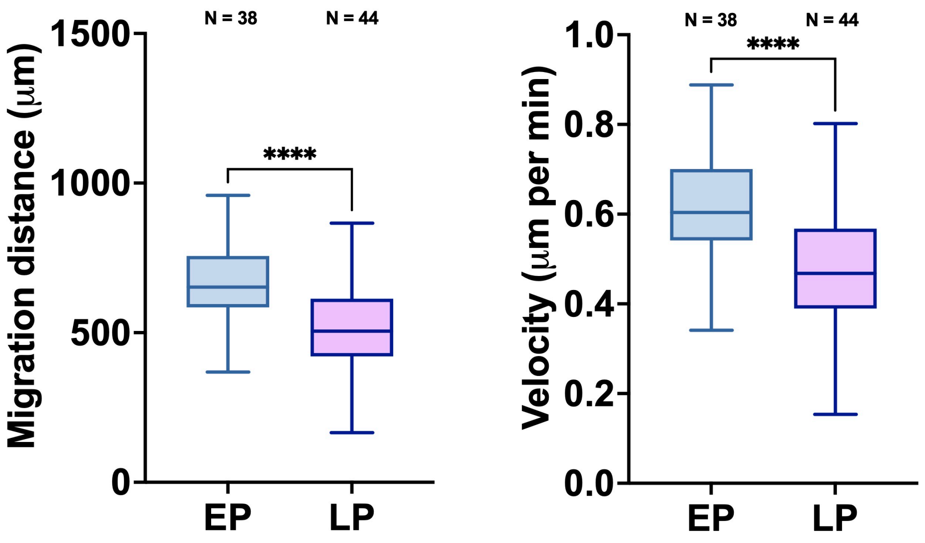

Figure S18 The accumulate migration distance and velocity of young and aged MSCs. The statistical differences are shown (**** $p < 0.0001$).

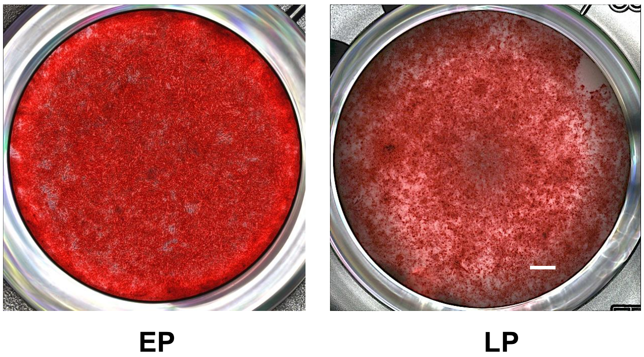

Figure S19 The Alizarin Red S staining of the young (EP) and aged (LP) MSCs after osteogenic induction. The scale bar is 500 μm.

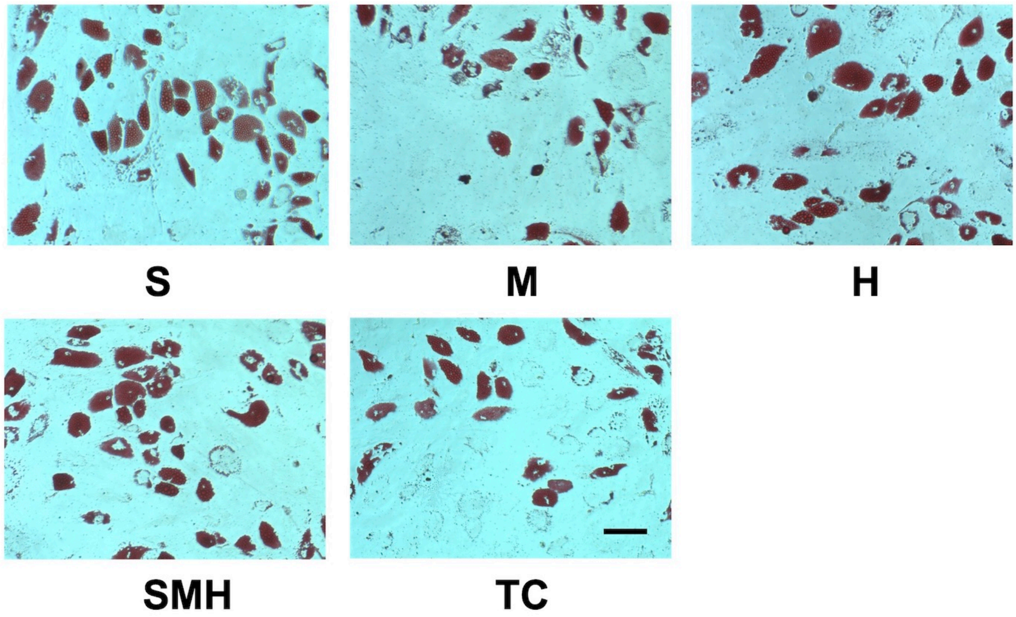

Figure S20 The Oil Red O staining of MSCs from gels and TC after adipogenic induction. The scale bar is 100 μm.

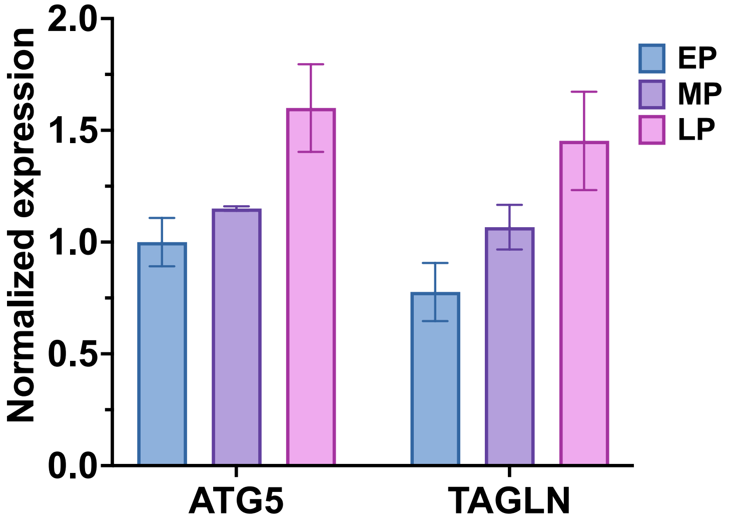

Figure S21 The expression of autophagy gene, ATG5 and transgelin (TAGLN) in young and RP MSCs.

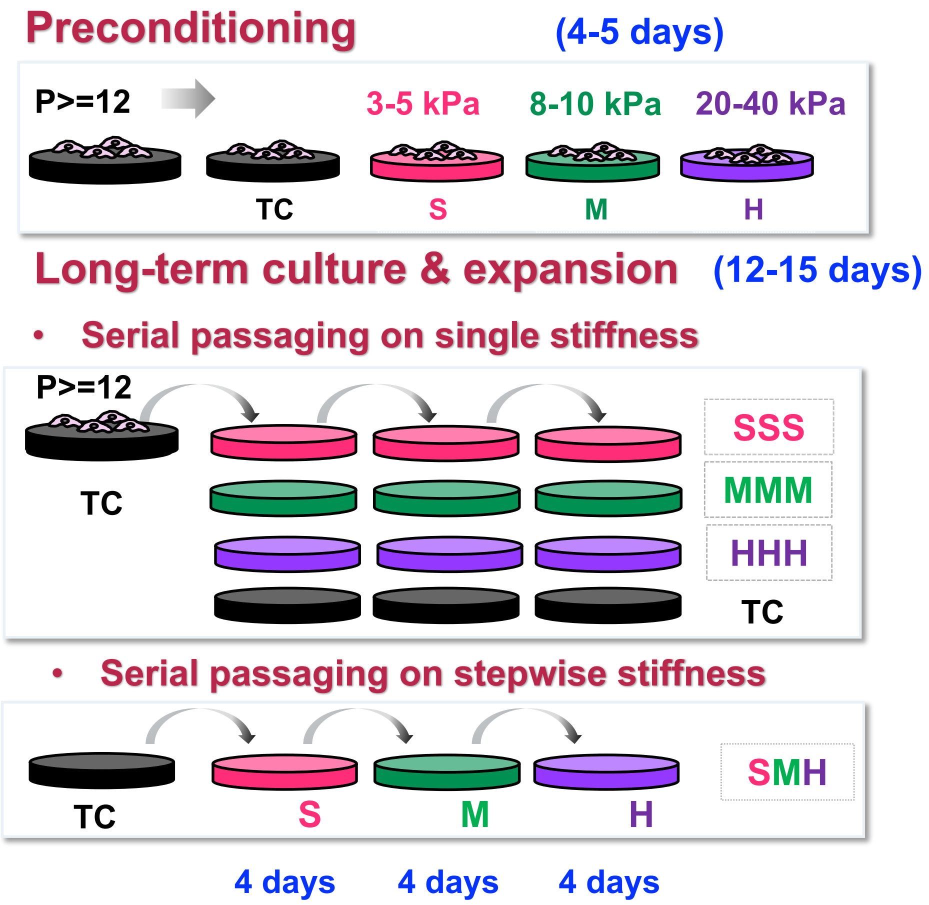

Figure S22 The schematic display of MSC expansion on surface elasticity tunable hydrogels. The MSCs were serially passaged on TC until reaching senescence state (P>=12). For preconditioning experiments, the LP MSCs were cultured on each gel substrate for 4 to 5 days. For long-term culture and expansion, the MSCs were seeded on the substrates for 4 days, then collected and reseed at the same density to the corresponding substrates for totally 3 cycles.
